# Nightjars trade off cruising speed efficiency for hovering capabilities and link upstroke thrust production with restricted wing folding

**DOI:** 10.1371/journal.pbio.3004023

**Published:** 2026-09-30

**Authors:** L. Christoffer Johansson, Gabriel Norevik, Sonja Friman, Anders Hedenström

**Affiliations:** Department of Biology, Lund University, Lund, Sweden; Chiba University, JAPAN

## Abstract

The European nightjar (*Caprimulgus europaeus*) is a long-distance migrant that also uses slow, hovering, flight as part of its feeding ecology. This reflects complex tradeoffs in aerodynamic mechanisms and makes studies of their flight performance, in relation to their wing shape, particularly interesting. Here we reveal, using wake visualization and aerodynamic measurements of nightjars flying in a wind tunnel, a unique double tip vortex phenomenon at cruising speeds. This correlates with reduced span efficiency at these flight speeds, suggesting relatively high costs of generating lift. Our interpretation is that the nightjar’s broad, slotted wing tip shape promotes lift during slow flight while incurring reduced aerodynamic efficiency at faster speeds. In addition, we found outer wing upstroke thrust generation during cruising flight—a characteristic previously attributed to bats and hummingbirds—in nightjars. This challenges an existing paradigm that considers an active upstroke to primarily add weight support in birds. Our kinematic analysis suggests this phenomenon may stem from constraints on wing folding during the upstroke, something that may apply generally to flapping flight. Our results highlight the tradeoffs seen in animals engaged in multiple costly behaviors with different aerodynamics optimization criteria based on ecological demands and offer potential solutions applicable in bioinspired engineering.

## Abstract 2

Birds such as nightjars must fly at different speeds during feeding and migration, but this versatility comes with trade-offs. This wind tunnel-based study identifies unexpected aerodynamic and kinematic flight features related to these ecological demands, and proposes a general link between limited wing folding and upstroke thrust generation.

## Introduction

The ability of active flight has opened novel niches and allowed for a range of ecologically relevant behaviors in birds, ranging from nectar feeding during hovering to long-distance migration. Coupled with different ecological behaviors, we find considerable diversity in the shape of wings and flight styles among bird species [1]. The combination of wing shape (morphology) and wing motion (kinematics) determines the aerodynamic performance—the magnitude, direction and energetics of force generation—of animals. How the combination of these factors drives the evolution of differences among species remains an important unresolved topic in biology. Variation in wing loading (weight/wing area) and wing shape (e.g. hand wing to wingspan ratio, aspect ratio—wing length/chord and chord distribution—how the width of the wing varies along the span) is often associated with differences in flight style among species [1,2], but each species may experience different flight demands, coupled to different behaviors, which could result in tradeoffs. By studying how individual species solve these conflicting demands, we may illuminate important clues to the relative costs of different solutions.

The aerodynamic/mechanical cost of flight depends on three components of power: parasite, profile, and induced power [3,4]. The first two power components relate to properties of the body and wings, respectively, and dominate at high flight (air) speeds, while induced power is related to the generation of lift that dominates at slow flight speeds [3,4]. The profile and induced power are, among other things, determined by the size and shape of the wings [4]. For a flapping wing, the relative size and shape that minimize profile or induced power are not necessarily aligned, inferring differently shaped wings for animals adapted for mainly flying slowly versus mainly flying fast [5]. However, in species that for ecological reasons need to, for example, fly across a wide range of speeds and modes, we may expect to find not only tradeoffs in wing morphology, but also different kinematic strategies related to aerodynamic performance. By studying species utilizing a wide range of flight speeds, we can improve our understanding of adaptations and constraints of animal flight. Here we provide results for one such species—the European nightjar.

The European nightjar (*Caprimulgus europaeus*) is an enigmatic bird species that stands out regarding the combination of wing size, shape, and flight style. This long-distance migrating crepuscular aerial insectivore rests motionless during the daylight hours, often on the ground where it also nests, while spending the twilight hours hawking insects [6–8]. This suggests a need to be able to fly across a wide speed range, flying slowly when hawking for insects (it is capable to hover without wind assistance) and to fly efficiently at faster speeds during migratory flights. These two feats are likely to favor different wing shapes and flapping motions [9,10], and combined with the rather unusual wing shape of nightjars (see below) suggest they provide an interesting species to study aerodynamically.

For a bird that is able to fly very slowly, and even hover for extended time, the nightjar is relatively heavy (71 g ± 11.7 g, S1 Table) [11]. Its wings are large and long for its mass [12], and relatively narrow with an aspect ratio of 7.5 (*AR* = *b*/*c*, where *b* is the wing span and *c* the mean chord. Aspect ratio typically ranges between 4 and 15 in birds [12]) (S1 Table). Unlike most birds with relatively high *AR* wings, their wingtips are comparatively broad (S1 Fig), which also differentiates the nightjar from many hovering specialists, such as hummingbirds [13]. Given the size of nightjars, it is likely that the broad wing tip is an adaptation to generate weight support at slow flight speeds by increasing the chord of the part of the wing that moves the fastest through the air [14]. The broad wing tips may, however, result in higher profile power (i.e., due to the friction drag), as well as increase induced power by creating a non-uniform downwash, reducing the span efficiency at higher speeds. Here we ask if there is an impact on the efficiency of flight at cruising speeds as a consequence of these apparent adaptations for slow flight.

To examine how the nightjar’s wing shape and wingbeat kinematics affect the aerodynamics, we measured the wake vortices and the power required to fly across a range of speeds. We determined the aerodynamic power from the wake and compared it with that predicted by a model for flapping flight [3]. We also determined several efficiency measures to evaluate how force production was affected across speeds and how the 3D kinematics changes accordingly.

## Results

We successfully captured data from three individuals ranging from 78 to 212 observations (flight sequences) depending on variable of interest; see S2–S18 Tables for details. The wake pattern varied across the studied flight speed range (2.6–11 m/s), particularly between the lowest speed (S2 Fig) and the other speeds. At cruising speeds (i.e., minimum power speed and faster) the wake showed undulating vortices, as expected for flapping flight, but with two distinct features not normally observed. We generally identified double tip vortices shed from the downstroke and reversed vortex loops formed at the outer part of the wings during the end of upstroke (Figs 1 and S3). The reversed vortex loops were associated with upwash and tilted in a way that indicates that the wing generates thrust (Figs 1 and 2a). At the lowest speed, there was one dominating tip vortex and sometimes weaker vortices proximal to the main vortex, which were often displaced by the induced flow (S2 and S4 Figs). At this speed, the upstroke wake was more complex than that of the downstroke. During the upstroke, the inner wing moved forwards relative to still air, while the hand wing moved backwards relative to still air due to a faster rearward flapping motion than the forward flight speed. As a result, the upstroke interacted with the wake of the previous downstroke, and the wing of the successive downstroke moved through the wake of the previous upstroke. This resulted in a wake that was difficult to interpret. Nevertheless, the outer wing clearly generated a vortex loop indicative of thrust production (i.e., with rearwards-directed induced flow) during the second half of the upstroke, when we observe a pitch-down “fanning” motion of the wing in the kinematics. Congruently, we observed rearwards induced flow (reflecting thrust production) shed at the outer wing during the majority duration of the upstroke. The kinematics indicated opposite-signed circulation on the inner and outer part of the wing during the upstroke, and in the videos, we noted a gap forming between the secondary feathers mid-wing. This led us to expect the occurrence of a vertically aligned vortex at this spanwise position, but no clear vertical vortex was present in the wake (S2 Fig). However, the wake of the inner wing was highly complex and variable during the upstroke (possibly due to high angles of attack) and the kinematic videos showed that the covert feathers were raised above the wing surface indicating low pressure and potentially separated flow with low circulation and high drag.

**Fig 1:**
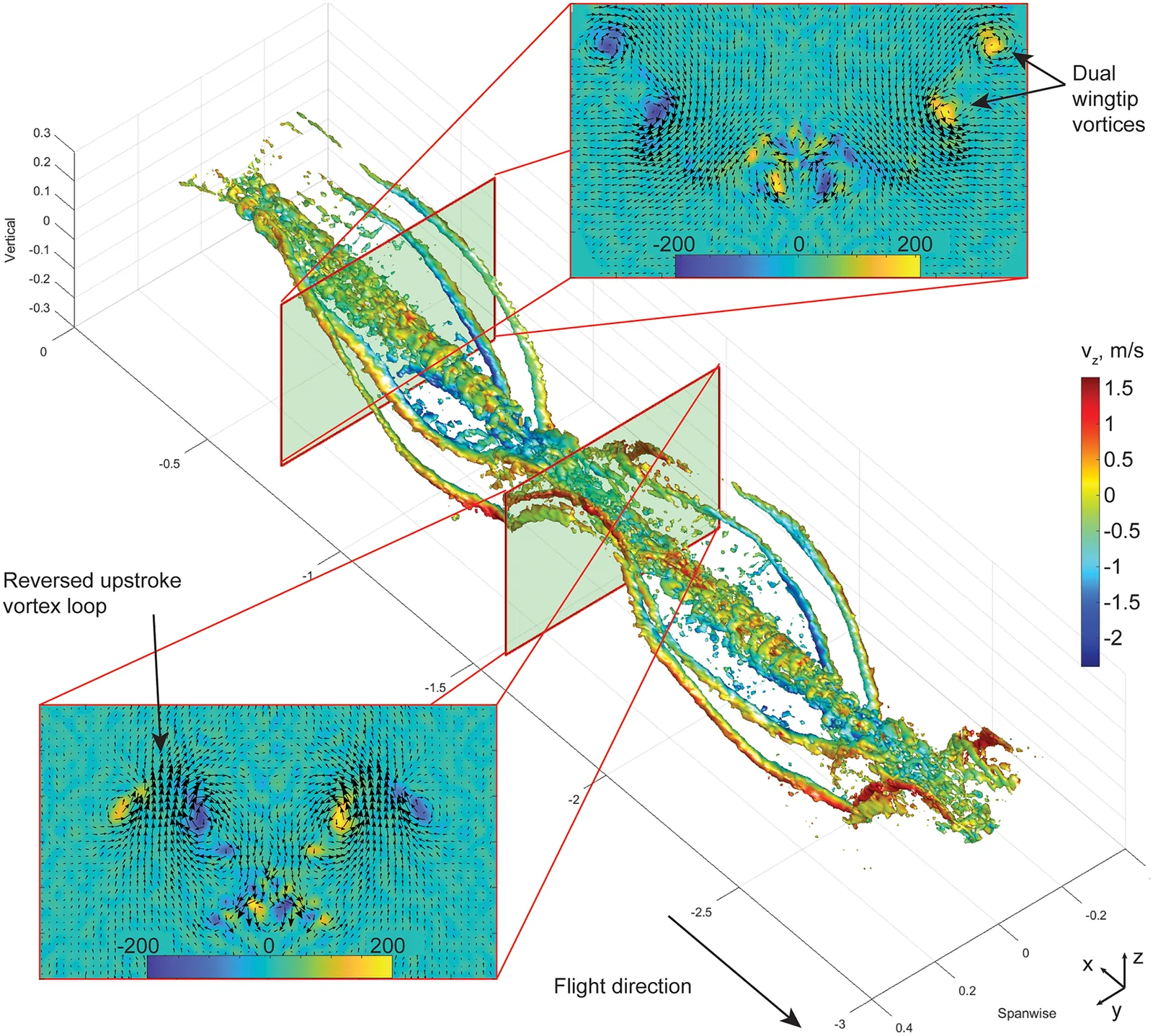
Nightjar vortex wake displays double tip vortices and upstroke thrust. Wake vortices shown as iso-surfaces of total vorticity [s^−1^] (iso value = 100), color-coded by downwash velocity (*v*_z_) of a sample sequence of a European nightjar flying at 9 m/s. Note the double tip vortices during the downstroke (upper inlet) and the reversed vortices at the outer wing during the upstroke (lower inlet). The inlets show in-plane velocity vectors on a background of the streamwise vorticity with blue showing clockwise rotation and yellow/red counterclockwise rotation. Vectors are not scaled the same in the two inlets but are scaled to facilitate interpretation of the flow. Units on the 3D-axes are in m.

**Fig 2:**
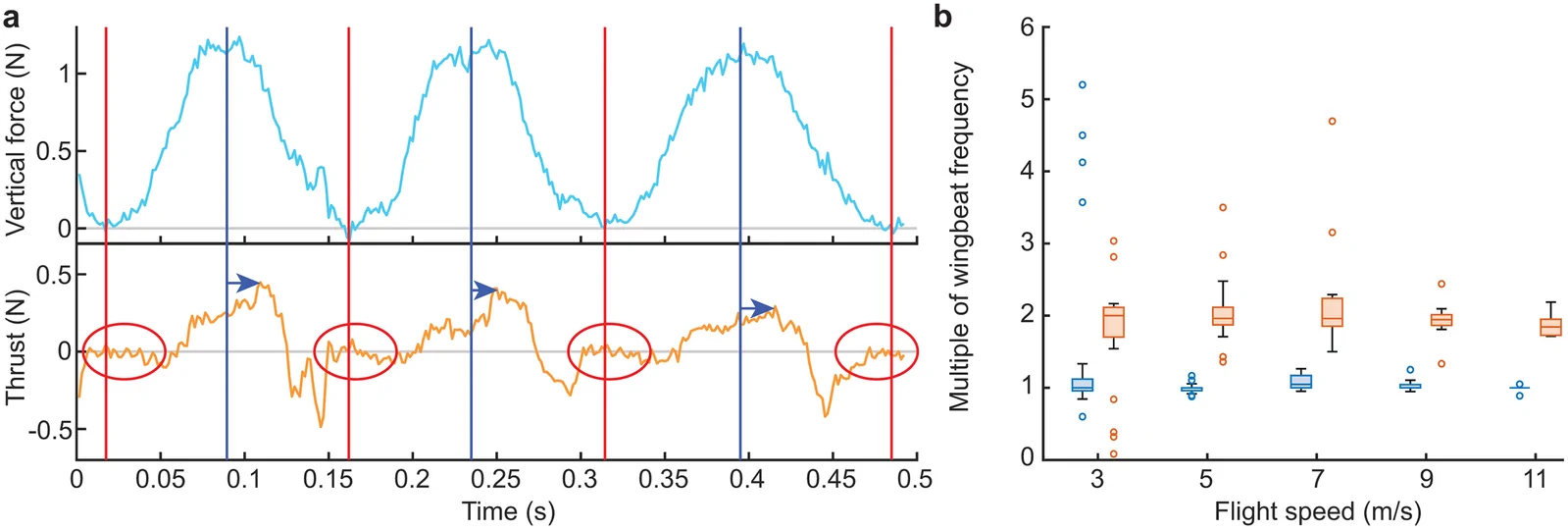
Forces estimated from wake measurements. **(a)** Sample sequence showing the vertical force (top) and thrust (bottom) over time. Vertical blue lines indicate the timing of peak vertical force, and vertical red lines indicate the timing of minimum vertical force. The minimum vertical force coincides with the lower thrust peak (minimum of negative thrust, encircled in red). The peak of the main thrust is delayed compared to the timing of the peak vertical force, as indicated by the blue arrows. Flight speed 9 m/s. **(b)** The multiple of the wingbeat frequency for the fft analysis of frequency from the thrust data across flight speed suggests dual thrust peaks during the wingbeat. The blue data shows the dominant frequency of the thrust signal divided by the dominant frequency of the vertical force signal (i.e., wingbeat frequency). The orange data show the frequency of the second peak of the thrust signal divided by the dominant frequency of the thrust signal. Data used to produce this figure can be found in S1 Data.

Across speeds, the downstroke provided the majority of the vertical force (*F*_v_), with the peak thrust (*T*) being delayed compared to the timing of the peak vertical force (Fig 2a). The minimum vertical force during a wingbeat occurred during the upstroke, coinciding with a second thrust peak (or minimum of negative thrust) (Fig 2a). The second strongest peak in the Fourier analysis of thrust across time had on average ~45% of the power of the strongest peak and clustered at 2 times the frequency of the strongest peak (i.e., the wingbeat frequency) across all speeds (Fig 2b). These results showed that the thrust often had two peaks during the wingbeat.

The power (*P*), normalized by the vertical force (*F*_V_), varied between 0.44 and 1.13 W/N and followed a U-shaped curve across speeds ($\frac{P}{F_{v}}=\frac{\text{0}.\text{9}\text{0}}{U_{\infty }}+\text{3}.\text{9}\text{2}{\text{1}\text{0}}^{-\text{5}}U_{\infty }^{\text{3}}+\text{0}.\text{5}\text{2}\text{4}$, for statistical output see S2 Table), with a minimum power of 0.65 W/N close to 9.3 m/s (Fig 3a). The curve follows a rather shallow U-shape compared to the model curve, with lower-than-predicted power at both the lowest and highest speeds (Fig 3a).

**Fig 3:**
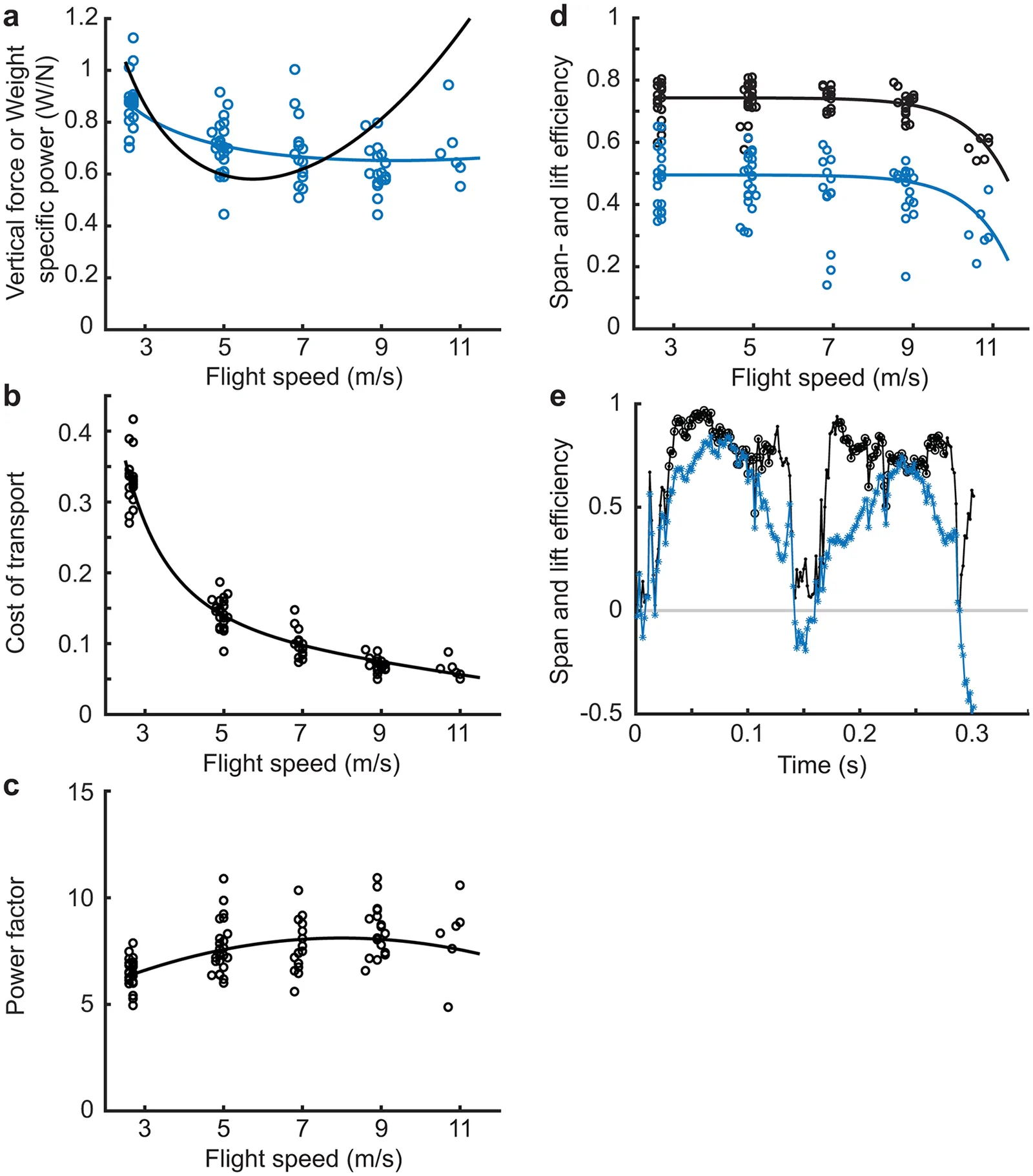
Flight efficiency increases with flight speed, but lift production becomes less efficient. **(a)** The lift-specific power across flight speed. Each dot represents the mean value for a sequence, and the fitted curve (blue) shows a U-shaped pattern with a minimum close to 9.3 m/s. The black curve is the expected weight-specific power curve, estimated using the Animal Flight Performance Tool (afpt, in R) [3]. Data used to produce this figure (i.e., Power and Mean lift) can be found in S1 Data. **(b)** The cost of transport (COT) decreases systematically with flight speed, indicating more efficient flight at higher speeds. Data used to produce this figure can be found in S1 Data. **(c)** The power factor (PF) increases with increasing flight speed with a shallow peak around 9 m/s, indicating that the overall force production is more efficient at higher than at lower speeds. Data used to produce this figure can be found in S1 Data. **(d)** Span efficiency (black) and vertical force efficiency (blue), during the downstroke, decrease with flight speed. This indicates that the efficiency of lift generation is lower at higher speeds than at lower speeds. The lower vertical force efficiency compared to span efficiency indicates efficiency loss due to generation of side forces that cancel between left and right wing and do not contribute to weight support. Circles represent median values during the downstroke. Data used to produce this figure can be found in S1 Data. **(e)** Span efficiency (black) and vertical force efficiency (blue) varies over a downstroke. Span efficiency varies relatively little during the downstroke (circles and dots), while vertical force efficiency shows a peak during mid-downstroke. The graph shows a sample of two wingbeats of a European nightjar flying at 7 m/s.

The two efficiency measures, cost of transport (*COT*) and power factor (*PF*), both showed an increasing efficiency with increasing flight speed ($COT=\frac{\text{1}.\text{7}\text{8}}{U_{\infty }^{\text{2}}}+\text{2}.\text{7}\text{6}{\text{1}\text{0}}^{-\text{4}}U_{\infty }^{\text{2}}+\text{0}.\text{0}\text{7}\text{5}\text{0}$, S3 Table, Fig 3b), ($PF=\text{4}.\text{2}\text{4}+\text{0}.\text{9}\text{6}\text{9}U-\text{0}.\text{0}\text{6}\text{0}\text{6}U_{\infty }^{\text{2}}$, S4 Table, Fig 3c) with a potential maxima at ~8 m/s. Span efficiency (*e*_i_), on the other hand, had a mean value of 0.72 during the downstroke and decreased with increasing flight speed ($e_{i}=\text{0}.\text{7}\text{4}\text{3}-\text{2}.\text{7}\text{2}{\text{1}\text{0}}^{-\text{6}}e^{U_{\infty }}$, S5 Table, Fig 3d). The vertical force efficiency, *e*_iFV_, was lower (0.45) than *e*_i_ and decreased with increasing speed ($e_{iFv}=\text{0}.\text{4}\text{9}\text{5}-\text{2}.\text{7}\text{9}{\text{1}\text{0}}^{-\text{6}}e^{U_{\infty }}$, S6 Table, Fig 3d). Within a single downstroke, span efficiency remained relatively constant, while the vertical force efficiency varied and peaked mid-downstroke. (Fig 3e).

Adjustment of the thrust-to-weight support ratio, as needed when changing flight speed, can be achieved in several ways. The kinematic analyses (S5 Fig) showed that the nightjars adjusted body tilt as well as stroke plane angle with flight speed. As flight speed decreases and generating weight support becomes increasingly challenging, requiring a more horizontally directed wing stroke, the birds tilted up their body ($Body\;tilt\;angle=\text{5}\text{5}.\text{1}-\text{8}.\text{2}\text{9}U_{\infty }+\text{0}.\text{4}\text{2}\text{2}U_{\infty }^{\text{2}}$, Fig 4a and S7 Table). This increased the angle of attack of the body but also helped tilt the stroke plane. The stroke plane angle became less vertical at low compared to high speeds ($Stroke\;plane\;angle=\text{1}\text{1}.\text{3}+\text{1}\text{2}.\text{5}U_{\infty }-\text{0}.\text{5}\text{6}\text{2}U_{\infty }^{\text{2}}$, Fig 4a and S8 Table) and changed more than the body tilt angle (Fig 4a). We found two additional changes indicative for an increased weight support at low speeds; an increased tail tilt ($Tail\;tilt\;angle=\text{2}\text{5}.\text{1}-\text{6}.\text{3}\text{1}U_{\infty }+\text{0}.\text{3}\text{5}\text{6}U_{\infty }^{\text{2}}$, S6b Fig and S9 Table) and tail spread angle ($Tail\;spread\;angle=\text{1}\text{0}\text{4}-\text{5}.\text{2}\text{3}U_{\infty }$, S6c Fig and S10 Table).

**Fig 4:**
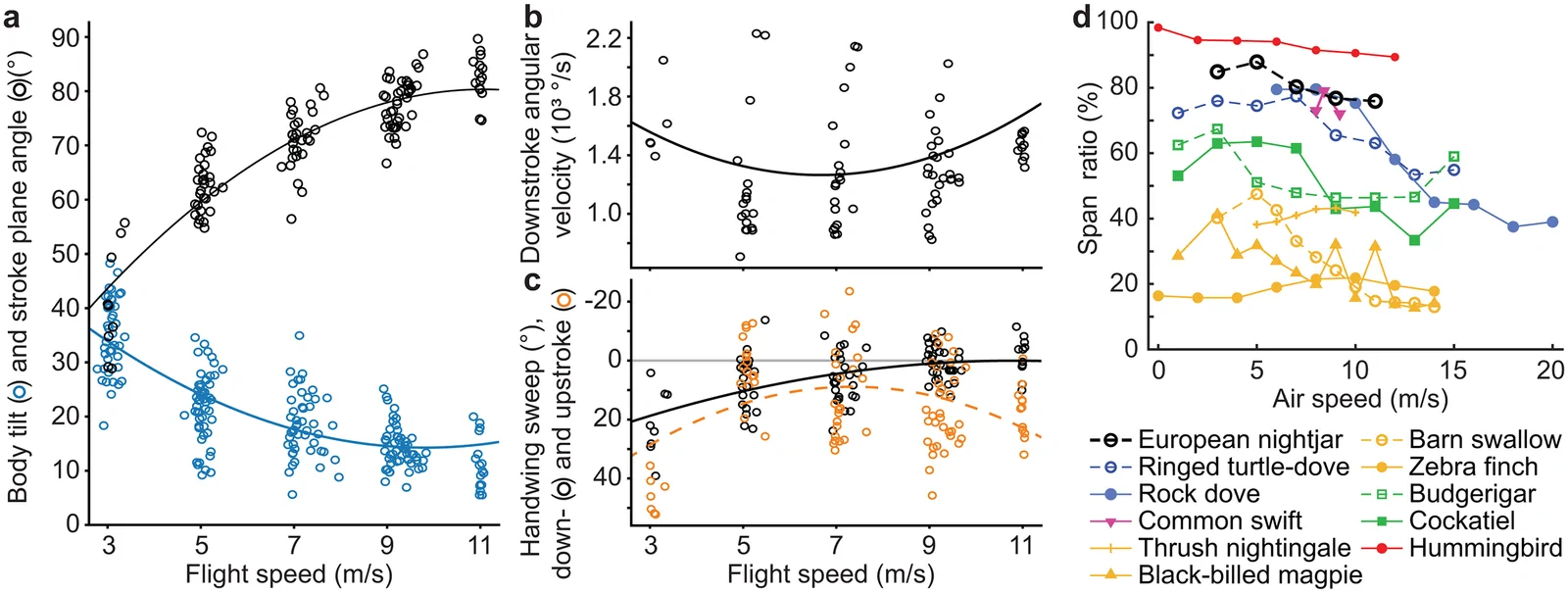
Kinematics vary with flight speed. **(a)** The birds tilt their body angle as well as their stroke plane angle as speed decreases. Data used to produce this figure can be found in S1 Data. **(b)** Downstroke angular velocity controls the forces generated by the wing, showing a U-shaped pattern with flight speed. Data used to produce this figure can be found in S1 Data. **(c)** Hand wing sweep (0 is perpendicular to the flight direction) varies relatively little across speeds and between down- and upstroke. Data used to produce this figure can be found in S1 Data. **(d)** Nightjars show a high span ratio across flight speeds compared to other bird species. The nightjar has a span ratio of around 80%, which is in the same range as in swifts and slightly below hummingbirds. Data from other species are digitized from figures in [15] and [16]. Raw data for span efficiency can be found in S6a Fig.

The effort to produce forces is related to the flapping velocity of the wing. When going from the lowest to the highest speeds we observe an increase in the flapping amplitude (~37%) ($Stroke\;angle\;amplitude=\text{6}\text{9}.\text{2}+\text{3}.\text{7}\text{1}U_{\infty }$, S6d Fig and S11 Table), but at the same time a decrease in the flapping frequency (~16%) ($Wingbeat\;frequency=\text{8}.\text{2}\text{4}-\text{0}.\text{1}\text{5}\text{9}U_{\infty }$, S6e Fig and S12 Table), while the downstroke ratio remained relatively constant ($Downstroke\;ratio=\text{0}.\text{5}\text{2}\text{7}-\text{0}.\text{0}\text{0}\text{3}\text{0}\text{8}U_{\infty }$, S6f Fig and S13 Table). Taken together, this represents a *U*-shaped variation in the flapping velocity over the studied speed range ($Mean\;downstroke\;anglular\;velocity=\text{2}\text{2}\text{3}\text{5}-\text{2}\text{9}\text{1}U_{\infty }+\text{2}\text{1}.\text{8}U_{\infty }^{\text{2}}$, Fig 4b and S14 Table), with a minimum close to 6.7 m/s flight speed.

Span ratio is a measure of how much the wing is retracted during the upstroke compared with downstroke. The span ratio was high (~80%), with a slight convex curve across speeds ($Span\;ratio=\text{0}.\text{9}\text{2}\text{5}-\text{0}.\text{0}\text{1}\text{8}\text{4}U_{\infty }$, Figs 4d and S6a; S15 Table). In the nightjars the span ratio was mainly controlled by retraction of the arm wing while the sweep of the hand wing during the upstroke was small ($Sweep\;angle\;DS=\text{5}\text{4}.\text{1}+\text{6}.\text{6}\text{3}U_{\infty }-\text{0}.\text{3}\text{0}\text{6}U_{\infty }^{\text{2}}$, $Sweep\;angle\;US=\text{2}\text{4}.\text{8}+\text{1}\text{5}.\text{3}U_{\infty }-\text{1}.\text{0}\text{4}U_{\infty }^{\text{2}}$, Fig 4c; S16 and S17 Tables).

## Discussion

The specialized ecology and morphology of the European nightjar, combining slow flight aerial foraging and long-distance migration with large and long wings, is reflected in the aerodynamics of the species. The examined nightjars showed distinct features previously not described in bird wake studies [16–29]. First, we found dual, same-sign wing tip vortices at cruising flight speeds (Fig 1). This indicates deviation from the ideal elliptic lift distribution and associated uniform downwash along the span, resulting in variable downwash behind the wing, which is reflected by decreasing span efficiency as flight speed increases (Fig 3d). Second, we observed an upstroke wake reflecting thrust generation at the outer wing at cruising speeds, at the expense of negative vertical force generation (Figs 1 and 2a). This is similar to what has previously been described for bats [30], insects [31,32], and suggested for hummingbirds [33,34]. This propounds a general aerodynamic mechanism: when wing folding is limited and span ratio remains high, upstroke thrust emerges as a robust solution across birds, bats and insects. Both these findings reflect tradeoffs associated with flapping flight across a wide range of speeds with implications for our understanding of flight performance tradeoffs across animal groups as well as robots with limited ability to flex wings between down- and upstroke.

The generation of upstroke thrust in bats has been interpreted as an adaptation to overcome high drag (e.g., due to ears) [30,35]. This is an unlikely explanation in nightjars given their streamlined body and that birds generally are considered more efficient fliers than bats [36]. Compared to previously studied bird species [22–24], except swifts [16] and hummingbirds [34], nightjars have relatively long hand wings (S1 Fig). We propose that folding and extending the long hand wing during the upstroke will incur a fast-sweeping motion back and forth in the flight direction, which may impede efficiency through increased wing drag or by causing unpredictable local flow phenomena at the wing [37]. By limiting the sweep of the hand wing during the upstroke (Fig 4c), the span ratio will remain high, which was confirmed by the kinematic analysis (Fig 4d). Previously studied birds, with relatively shorter hand wings, have not exhibited any signs of upstroke thrust [20–23,25]. A robotic study representing an avian wing showed that upstroke wing folding increased the efficiency of force production [38]. Taken together, these findings suggest that there is a cost associated with no or minimal folding of the wings during the upstroke, but that this cost is less than the alternative of fully folding the wing in species like the nightjar. Interestingly, revisiting the wake of the common swift, *Apus apus* ([26], Fig 3n), we find a presence of reversed vortex loops (i.e., thrust production) during the upstroke at relatively high flight speeds, which is expected due to their relatively long hand wings and high span ratio (Fig 4d). In addition, computational fluid dynamics modeling and wake measurements suggest upstroke thrust also in hummingbirds [33,34]. Hence, we may expect that also other species with relatively long hand wings will use the same strategy as nightjars, swifts and hummingbirds.

If the presence of a high span ratio reflects a strategy to avoid detrimental flow phenomena related to wing folding in nightjars, the same may apply to other animals. In bats, maintaining a taut hand wing membrane may avoid high drag penalties (due to membrane flutter [39]), but result in a relatively high span ratio (~70%–90% [40–43]). Insects, having jointless wings, also exhibit (unavoidable) high span ratios (~100%). The best upstroke strategy in cruising flapping flight, when not retracting the wings fully, has traditionally been hypothesized to be to use a “constant circulation” throughout the wingbeat [4,27]. In this case, the upstroke is used to generate weight support and some negative thrust to maintain the direction and magnitude of the circulation around the wing, keeping the induced costs low. In contrast, animals that use an active upstroke have been shown to generate thrust (bats [30], insects [31,32] and birds (current study [26,32,33]). This, despite the increase in induced power resulting from the change of sign of circulation associated with the upstroke thrust [38]. A high span ratio will result in a relatively high upward velocity of the distal part of the wing. Unless the wing is pitched up substantially, this will unavoidably result in negative angles of attack and thrust production. At cruising speed, the upstroke thrust strategy may therefore reflect that the cost of generating weight support is relatively low. At cruising speed induced power constitutes a small portion of total power, while thrust requirements are high and dependent on an increase in flapping velocity if to be generated entirely during the downstroke. What we have found may thus be a general strategy reflecting how to best accommodate, for different reasons, a limited ability to fold the wing during the upstroke in flapping flight.

Birds tend to have higher span and/or flap efficiency compared to bats [36]. However, span efficiency during the downstroke in our nightjars is within the same range as that reported for bats using the same technique [44], while also showing a tendency to decrease at the highest flight speeds (Fig 3d). The dual wing tip vortices found in the nightjar wake (Fig 1) reflect a change in the circulation strength along the wingspan, and hence a non-uniform downwash distribution resulting in reduced span efficiency [45]. One mechanism that could cause a spanwise change in circulation, resulting in the second tip vortex, is the non-continuous decrease in the chord towards the wing tips [46]. Nightjars have an unusual wing shape, with the three distalmost primary feathers forming an almost square-shaped wing tip with separated feathers (S1 Fig). A broad, multi-slotted, wing tip has been suggested as an adaptation to generate high lift at slow speeds by providing an effective washout [14]. We observe formation of less prominent double tip vortices at the lowest flight speed, indicating that the nightjar wing shape may function better at low advance ratios (forward compared to flapping speed). This is also supported by the measured power being below model estimates at the lowest speeds (Fig 3c) and the low-speed span efficiency being higher than at cruising speeds (Fig 3d). However, a limitation of our method is that we measure in the wake of the animals and at low speed there is a possible wing-wake interaction (e.g., wake capture) that may reduce the wake kinetic energy. Future modeling or robotic studies are required to better understand under what conditions double wing tip vortices are generated in flapping flight and the possible effects of wing-wake interactions. It is worth noting that the higher the flight speed, the lower the fraction of the total power is accounted to induced power. This means that a flying animal could afford less efficient lift generation at higher speeds, without necessarily paying a high cost. For a species, such as the nightjar, reliant on slow flight capabilities in cluttered environments for food capture, while being relatively heavy, it may be beneficial to trade span- and lift efficiency at high speeds against a more effective (or efficient) force production at slow speeds. In addition, on long-distance flights, flight style may be changed to reduce the costs. It is interesting to note that nightjars have been observed to use flap gliding and to fly close to water surfaces during migration across large bodies of water [47], which may reduce the cost of flight [48].

It typically becomes more challenging to generate weight support as flight speeds decrease. We found that the nightjars responded to this by adjusting their body-tilt and stroke plane angles accordingly (Fig 4a). This results in the velocity gradient of the air meeting the wing being stronger along the span at slow compared with high flight speeds, which moves the center of the aerodynamic force distally on the wing. The long wings of the nightjars likely allow them to generate more aerodynamic force at slow flight speeds. A large and distal aerodynamic force increases the torque required to flap the wing and thereby the muscle force needed. Since the mechanical power of the muscles is the force × contraction velocity it is interesting that we find different flight speeds at the minimum for lift-specific mechanical power (Fig 4a) and the downstroke velocity (proportional to the muscle contraction velocity) (Fig 4b) since this suggests that the force required to flap the wing is higher at low compared to high flight speeds, supporting the notion that the wing morphology aids force production during slow flight.

To conclude, our analyses of the aerodynamic performance of the European nightjar have revealed flow features rarely or not previously observed in birds. This includes an active, thrust-producing upstroke and dual, similar-signed “tip” vortices (Fig 1). The latter reflects a relatively inefficient lift production at cruising speeds, supported by a decline in span efficiency with flight speed (Fig 3d), even though the species is a long-distance migrant [7]. At the same time, the nightjar is capable of slow flight, and even short periods of hovering, and our results suggest that the vertical force efficiency is relatively high at these slow speeds (Fig 3). We thus conclude that our findings represent an efficiency tradeoff between flying slowly and flying fast in a bird species with ecological demands ranging from hovering to long-distance migration. As such, the results question the general notion that long-distance migration always requires exceptional aerodynamic efficiency. In addition, the results highlight a potentially general aspect of flapping flight—an association between thrust production and a restricted wing folding during upstroke. These findings should affect our expectations for aerodynamic performance in animal flight across taxa and may inspire technical solutions to flapping flight.

## Materials and methods

### Ethics statement

The study was performed in accordance with the experimental procedures approved by the Malmö–Lund animal ethics committee (permit M 33-13), Sweden. After the completion of the study the birds were released.

### Animals

Five European nightjars (*Caprimulgus europaeus*) were captured in our long-term study area (16°E, 57°N) in summer 2016 and transported to our facility at Lund University (13.2°E, 55.7°N). The birds were trained to fly in a wind tunnel [49], by slowly extending their flight time after being released from a perch to when they were allowed to land on the perch again. Three birds, that repeatedly flew steadily for at least 30 seconds and were determined capable of flying at a range of speeds, were selected for the experiments, while the other two were released. The morphometrics of these individuals (S1 Table) are representative for the study population [11], and photos of the wing planforms can be found in the Supporting information (S1 Fig).

### Experimental setup

The animals were flown in a wind tunnel at preset speeds (*U*_∞_) ranging from 2.6 to 11 m/s in approximately 2 m/s intervals. The wind tunnel is a recirculating tunnel capable of speeds between 0 and 38 m/s, with a 12:1 contraction ratio and low turbulence levels [50]. The test section has an octagonal cross section, 1.2 m wide and 1.08 m high, with an open section behind the test section that allows for control of the animals. The birds were filmed in IR lighting using two high-speed cameras (LaVision Imager pro HS 4M, 2,016 × 2,016 pixels, LaVision, GmbH, Göttingen, Germany). The videos were analyzed for 3D kinematics (see below). To capture the flow induced by the birds, we used a standard stereo particle image velocimetry (sPIV) setup with a plane perpendicular to the wind tunnel flow, as previously described in [23]. The field of view was approximately 47 × 55 cm (*w* × *h*) with a vector resolution of 2.5 vectors per cm and a sampling frequency (*f*_s_) of 640 Hz. We defined a right-handed coordinate system with *x* in the downstream direction and *z* vertically upwards.

### PIV settings and flow field analyses

The PIV analysis was done in Davis 8 (LaVision, Göttingen, Germany). For details regarding the settings, see Supporting information. The final vector fields were stacked with a spacing equal to *U*_∞_*f*_s_ and if only the wake of one wing was visible, mirrored in the wake center plane. All vorticity components were then estimated using the *curl* function in Matlab. From the vector fields, we estimated the vertical force (weight support, *F*_V_) and net thrust (*T*) in each frame, over a discrete number of wingbeats in each sequence, following the descriptions in [51]. Hence, vertical force at each time step was estimated as:


$$\begin{array}{c} F_{V}=\rho U_{\infty }\iint_{wakearea}\left(y-y_{\text{0}}\right)\cdot \omega_{x}(y,z)dydz, \end{array}$$ (1)

where *ρ* is the air density, *y*-*y*_0_ the horizontal distance to the center of the wake and *ω*_x_ the streamwise vorticity.

Net thrust was similarly estimated from the vorticity field at each time step as:


$$\begin{array}{c} T=\frac{\text{1}}{\text{2}}\rho U_{\infty }\iint_{wakearea}\left(y-y_{\text{0}}\right)\cdot \omega_{z}(y,z)-\left(z-z_{\text{0}}\right)\cdot \omega_{y}(y,z)dydz, \end{array}$$ (2)

where *z* − *z*_0_ is the vertical distance to the center of the wake, *ω*_y_ is the spanwise vorticity and *ω*_z_ is the vorticity along the vertical axis.

We also estimated the power (*P*) from the rate of kinetic energy (*E*) added to the wake by the bird [52] following the procedure in [53], where the wake is “auto-masked” to isolate the vorticity found in the wake to reduce the effect of noise in the background flow. The wake was then extended to a cross-sectional size of 2.4 × 2.4 m, using the Helmholtz–Hodge decomposition [52] before estimating the kinetic energy in the wake.


$$\begin{array}{c} E=\frac{\text{1}}{\text{2}}\rho {∭}_{Wakevolume}{(u}^{\text{2}}+v^{\text{2}}+w^{\text{2}})\cdot \left(\text{1}+\frac{u}{U_{\infty }}\right)dxdydz, \end{array}$$ (3)

$$\begin{array}{c} P=\frac{E}{N_{wb}}f, \end{array}$$ (4)

where *u*, *v* and *w* are the induced velocities (the change in velocities relative to still air caused by the animals, i.e., subtracting *U*_∞_ from the measured velocity) in the *x*, *y*, and *z* directions, respectively, *N*_wb_ is the number of wingbeats in the sequence and *f* is the wingbeat frequency.

In addition to the above-mentioned outputs, we estimated several efficiency measures. We calculated the *COT*, a measure of the energy required to move one Newton of weight (*W*) the distance of one meter as,


$$\begin{array}{c} COT=\frac{P}{F_{V}U_{\infty }}, \end{array}$$ (5)

where *F*_V_ should ideally equal *W* (see below regarding selection of data). We also estimated an additional efficiency measure, the *PF*, originating from actuator disk models [54,55], as


$$\begin{array}{c} PF=\frac{C_{F}^{\text{1}.\text{5}}}{C_{P}}, \end{array}$$ (6)

where *C*_F_ and *C*_P_ are the force and power coefficients, respectively. These coefficients were calculated as


$$\begin{array}{c} C_{P}=\frac{P}{qU_{\infty }S}, \end{array}$$ (7)

$$\begin{array}{c} F={{\left((\frac{P}{U_{\infty }}\right)}^{\text{2}}+F_{V}^{\text{2}})}^{\text{0.5}}, \end{array}$$ (8)

$$\begin{array}{c} C_{F}=\frac{F}{qS}, \end{array}$$ (9)

where *q* is the dynamic pressure ($\text{0}.\text{5}\rho U_{\infty }^{\text{2}}$), *S* is the wing area and *F* is the mean force generated during a wingbeat. *F* was calculated as the vector sum of the thrust required to fly (*T*_R_ = -total drag (= *P*/*U*_∞_)) and *F*_V_.

We also estimated two versions of span efficiency, a measure of how variable the induced flow is along the span relative to the ideal uniform flow, during the downstroke. The first measure, which we call span efficiency (*e*_i_), examines the flow perpendicular to a line connecting the center of the wing tip vortex and the center of the body wake. The second measure we term vertical force efficiency (*e*_iFV_), which compares the measured induced flow to the ideal vertically induced flow needed to generate the same amount of weight support. For the *e*_i_ and *e*_iFV_ measurements, we developed an automatic tracking routine that, after performing a Gaussian smoothing (Matlab function *smoothdata2* with settings “gaussian” and box size 21) of the streamwise vorticity field (*ω*_x_), determined the 3–5 dominant peaks of vorticity in the wake on one side of the body and used the product of the vorticity and the square of the distance between the vortex and the center of the wake to find the distal-most dominant vortex, which defined the wing tip vortex. The induced flow, perpendicular to the line between the wing tip vortex and the center of body, was then interpolated at equidistant positions along the line (Matlab, *scatteredInterpolant*). The force perpendicular to the line, as well as the induced power (*P*_i_), were estimated following [44]. The ideal power (*P*_ii_) was then calculated by estimating the uniform induced velocity that would generate the same force. *e*_i_ was then determined by dividing the *P*_ii_ with *P*_i_. To estimate *e*_iFV_ we determined the ideal power to generate the same amount of weight support (*P*_iiFV_), by multiplying the ideal uniform flow, described above, with the cosine of the angle of the center of body to vortex line relative to the horizontal plane. *e*_iFV_ was then determined by dividing the *P*_iiFV_ with *P*_i_.

### Kinematics

To determine the kinematics, we trained (for 420,000 iterations with a final test error of 9.92 px) a deep neural network using *DeepLabCut* [56] to track 17 points on the left wing, body and tail of the birds (tip of the bill, wing root, wrist, center of the leading edge of the hand wing (*cle*), the tips of the three distalmost primary feathers, four points on the trailing edge of the wing, tail base, three points at the distal edge of tail (rightmost, center and leftmost) and each foot) in two separate views (S7 Fig). The 2D data were then filtered to remove outliers using the built-in filter function in DLC by thresholding the detection likelihood (>0.8) and making sure the points were not at the edge of the image. The data was then smoothed using a cubic spline over time, where points deviating more than 3 standard deviations from the mean deviation were removed. After interpolating missing points and gaps (less than 30 frames) using a smoothing spline, we triangulated the points from the two camera views, to get the 3D position of the points in each frame and removed points with an RMS > 20 mm. Calibration of the cameras was performed using “wand calibration” with the MATLAB tools DLTdv8a and simpleWand 0.8.5 [57]. For kinematics, a coordinate system with *x* pointing upstream, *y* crosswind and *z* vertically was defined. Flight velocity was determined as the velocity of the bill along *x* plus the wind tunnel velocity.

Using coordinates of the points, we then determined body tilt angle (angle between the line connecting the wing root and the tail base and the horizontal plane in the *xz*-plane), tail tilt angle (angle between the line connecting the tail base and the center tail feather tip and the horizontal plane in the *xz*-plane) and tail spread angle (between the lines connecting the tail tips and the tail base, assuming a symmetrical tail spread as the right tail base was not visible in the cameras). We determined the stroke plane angle (the tilt of the direction of maximum amplitude of the *cle*’s sweeping path relative to the shoulder in the *xz*-plane, relative to the *x*-axis, S7b Fig). The angular amplitude (*A*) of the *cle* was determined as the angle swept projected onto the stroke plane. We determined the wingbeat frequency (*f*) using a Lomb Scargle periodogram on the vertical component of the vectors between 7 points on the wing and the wing root, taking the average of the detected frequencies. We determined the downstroke ratio (*dsr*) as the quotient of the mean downstroke duration and the mean duration of a wingbeat by determining the timing of the extremes of the vertical wing locations. Using the amplitude, frequency and downstroke ratio, we calculated the downstroke angular velocity as AngVel=Af/dsr. In addition, we determined the mean wing elevation (*MWE*) of the *cle* [51], as the average of max 10% and min 10% angles. Mid-downstroke and mid-upstroke were then defined as when the wing was at the *MWE*. Span ratio is defined as the average upstroke wingspan divided by the average downstroke wingspan (during mid-upstroke and mid-downstroke). Hand wing sweep angle was defined as the angle of a line connecting the wrist and the *cle* relative to the *xz*-plane.

### Modelling flapping power

For comparison with measurements, we determined the predicted power required for flapping flight in European nightjars using the Animal Flight Performance Tool (*afpt*, in R [58],) as described in [3]. In the model, we used the average weight, wing span and area of the birds presented in S1 Table. In addition, we fitted a linear regression of the flapping frequency, *f*, against $U_{\infty }$ and included the estimated *f* in the model.

### Data selection and statistics

For the PIV data, we required the sequence-averaged vertical force (*F*_V_) to be within ±20% of the weight of the animal to be considered steady flight (i.e., not climbing or descending) (S8 Fig) and sequences that fell outside that range were excluded from further analyses, as in previous studies of animal flight [25,26]. The number of wingbeats in each sequence was not constant (varied between 1 and 7) and the number of sequences differed between individuals (“Left”, *N* = 24; “Right2”, *N* = 39; “Alfa”, *N* = 23).

To describe how the variables of interest varied across speed, while taking repeated measures from the different individuals into account, we fitted a mixed linear model to the data in Matlab using the *fitlme* function with REML as fit method. For these analyses, individual was set as a random factor, with interaction effects on intercept as well as on the continuous variables (except for span and lift efficiency and kinematic variables). *N*_wb_ in each sequence was used as weight in the model. The significance level was set to 0.05. Model assumptions were assessed graphically, by inspection of residuals versus fitted values, residual histograms and normal probability plots of residuals. Although several models showed moderate deviations from normality in the tails, residuals were generally centered around zero and given the general insensitivity of mixed linear models to violations of assumptions [59] the models were considered acceptable for describing speed-dependent trends while accounting for repeated measurements within individuals.

We fitted a mixed linear model with *P*/*F*_V_ as the dependent variable and included ${\text{1}/U}_{\infty }$ and $U_{\infty }^{\text{3}}$ as continuous variables following theoretic aerodynamical expectations [4].

We fitted a mixed linear model with COT as dependent and with $U_{\infty }^{-\text{2}}$ and $U_{\infty }^{\text{2}}$ as independents since COT is expected to follow the same relation with speed as drag [4]. We also fitted a mixed linear model with PF as dependent and with $U_{\infty }$ and $U_{\infty }^{\text{2}}$ as independent variables.

We fitted a mixed linear model with span (*e*_i_) and vertical force (*e*_iFv_) efficiencies as dependents and with an *exp* transformation of speed as independent. The transformation of speed was done to better fit the linear requirements of the model.

To examine the presence of thrust production during the upstroke, as well as during the downstroke, we performed a Fourier analysis (Matlab, *fft* function) of *F*_V_ and *T* as functions of time, following smoothing using a sliding average of length 7. We then compared the frequency with the highest power to the frequency with the second highest power for T to that of *F*_v_.

To describe how the kinematic parameters varied with speed, while accounting for the repeated measures, we fitted a mixed linear model to the data, using the individual kinematic variables as dependent variable and included $U_{\infty }$ and $U_{\infty }^{\text{2}}$ as continuous variables. Individual was set as a random factor, with interaction effects on intercept as well as on the continuous variables. Since we did not have an expected function for how the variables should vary with speed, we tested both linear and quadratic models and present the model with the lowest AIC (Akaike Information Criterion) [60]. We refrained from using higher-order models, to avoid overfitting, even if results indicated that the models did not fully capture the observed variation and as a consequence the results should only be seen to describe general trends in the data, rather than functional relationships.

## Supporting information

S1 Text Additional Material and methods describing details of the PIV setup and further Results and discussion [61]. (PDF)

S1 Fig Photos showing the wings of the nightjars used in this study, alfa (top), left (center) and Right2 (bottom). (PDF)

S2 Fig Wake vortices shown as iso surfaces of total vorticity [s^−1^] (iso value = 150), colored by downwash velocity of a sample sequence of a European nightjar flying at 2.6 m/s. Note the complex wake during the “upstroke” (lower inlet), cutting through two successive downstrokes and one upstroke and the weak flow in the body region during the downstroke (upper inlet). The inlets show in-plane velocity vectors on a background showing the streamwise vorticity with blue showing clockwise rotation and yellow/red counterclockwise rotation. Vectors are not scaled the same in inlets but are scaled to facilitate interpretation of the flow. Axes units are in m.
(PDF)

S3 Fig Isosurfaces of the total vorticity [s^−1^] (iso value 100) of the wake of a nightjar flying at 9 m/s. Oblique view (**a**), side view (**b**) and top view (**c**). The wake is mirrored in the center of the body and the bird is flying to the right. Axes units are in m.
(PDF)

S4 Fig Isosurfaces of the total vorticity [s^−1^] (iso value 150) of the wake of a nightjar flying at 2.6 m/s. Oblique view (**a**), side view (**b**) and top view (**c**). The wake is mirrored in the center of the body and the bird is flying to the right. Axes units are in m.
(PDF)

S5 Fig Sample sequences of wingbeat motions across speeds in European nightjars. Tracks over frames for the wing tip (red), trailing edge (green), wrist (orange) relative to the shoulder (blue) as seen from the front (left column), the side (middle column) and below (right column), going from slow (top row) to fast flight (bottom row). Note the change in mean wing elevation across speed and the limited span ratio in the left column and the change in stroke plane in the middle column.
(PDF)

S6 Fig Kinematics vary with flight speed. Nightjars show a high span ratio across flight speeds (**a**). The birds increased the tilt of the tail relative to the horizontal plane (tail tilt angle, [degrees]) (**b**) and tail area (tail spread angle, [degrees]) (**c**) at lower compared to higher speeds. Angular amplitude [degrees] increases with speed (**d**), while wingbeat frequency [Hz] decreases (**e**). Downstroke ratio is constant across flight speed (**f**). Mean wing elevation decreases [degrees] with increasing flight speed (**g**). For the statistics associated with the fitted lines, we refer to S7–S19 Tables. Data used to produce this figure can be found in S1 Data.
(PDF)

S7 Fig Definition of the morphological points tracked for the kinematic analysis of nightjar flight (a). The two points on the feet are not seen in this view. (**b**) Definition of kinematic angles; stroke plane (*β*), body tilt angle (*γ*), angular amplitude (*A*_ang_) and mean wing elevation (MWE).
(PDF)

S8 Fig Estimated weight support for the different individuals across flight speeds. Asterisk, individual “Left”; circle, “Right2”; triangle, “Alfa”. Each point represents the mean value for one sequence (1–7 wingbeats). Data used to produce this figure can be found in S1 Data.
(PDF)

S1 Table Morphometric information for the three European nightjars included in the analysis. (PDF)

S2 Table Statistical model results for P/F_v_ (Y) across speeds. Model assumes constant profile power, induced power varying with 1/U (X1) and parasitic power varying with U^3^ (X2). Individual (X4) is included as a random effect and data are weighed by number of wingbeats in the sequence. Number of observations refers to the number of recorded flight sequences.
(PDF)

S3 Table Statistical model results for COT (Y) across speeds. Model assumes constant profile drag, induced drag varying with 1/U^2^ (X1) and parasitic drag varying with U^2^ (X2). Individual (X4) is included as a random effect and data are weighed by number of wingbeats in the sequence. Number of observations refers to the number of recorded flight sequences.
(PDF)

S4 Table Statistical model results for PF (Y) across speeds. Model consists of intercept, U (X1) and U^2^ (X2). Individual (X4) is included as a random effect and data are weighed by number of wingbeats in the sequence. Number of observations refers to the number of recorded flight sequences.
(PDF)

S5 Table Statistical model results for e_i_ (Y) across speeds. Model consists of intercept and exp(U) (X1). Individual (X4) is included as a random effect and data are weighed by number of wingbeats in the sequence. Number of observations refers to the number of recorded flight sequences.
(PDF)

S6 Table Statistical model results for e_Fv_ (Y) across speeds. Model consists of intercept and exp(U) (X1). Individual (X4) is included as a random effect and data are weighed by number of wingbeats in the sequence. Number of observations refers to the number of recorded flight sequences.
(PDF)

S7 Table Statistical model results for Body tilt angle (Y) across speeds. Model consists of intercept, U (X1) and U^2^ (X2). Individual (X4) is included as a random effect. Number of observations refers to the number of recorded flight sequences.
(PDF)

S8 Table Statistical model results for Stroke plane angle (Y) across speeds. Model consists of intercept, U (X1) and U^2^ (X2). Individual (X4) is included as a random effect. Number of observations refers to the number of recorded flight sequences.
(PDF)

S9 Table Statistical model results for Tail tilt angle (Y) across speeds. Model consists of intercept, U (X1) and U^2^ (X2). Individual (X4) is included as a random effect. Number of observations refers to the number of recorded flight sequences.
(PDF)

S10 Table Statistical model results for Tail spread angle (Y) across speeds. Model consists of intercept and U (X1). Individual (X4) is included as a random effect. Number of observations refers to the number of recorded flight sequences.
(PDF)

S11 Table Statistical model results for Stroke angle amplitude (Y) across speeds. Model consists of intercept and U (X1). Individual (X4) is included as a random effect. Number of observations refers to the number of recorded flight sequences.
(PDF)

S12 Table Statistical model results for Wingbeat frequency (Y) across speeds. Model consists of intercept and U (X1). Individual (X4) is included as a random effect. Number of observations refers to the number of recorded flight sequences.
(PDF)

S13 Table Statistical model results for Downstroke ratio (Y) across speeds. Model consists of intercept and U (X1). Individual (X4) is included as a random effect. Number of observations refers to the number of recorded flight sequences.
(PDF)

S14 Table Statistical model results for Mean downstroke angular velocity (Y) across speeds. Model consists of intercept, U (X1) and U^2^ (X2). Individual (X4) is included as a random effect. Number of observations refers to the number of recorded flight sequences.
(PDF)

S15 Table Statistical model results for Span ratio (Y) across speeds. Model consists of intercept and U (X1). Individual (X4) is included as a random effect. Number of observations refers to the number of recorded flight sequences.
(PDF)

S16 Table Statistical model results for Sweep angle DS (Y) across speeds. Model consists of intercept, U (X1) and U^2^ (X2). Individual (X4) is included as a random effect. Number of observations refers to the number of recorded flight sequences.
(PDF)

S17 Table Statistical model results for Sweep angle US (Y) across speeds. Model consists of intercept, U (X1) and U^2^ (X2). Individual (X4) is included as a random effect. Number of observations refers to the number of recorded flight sequences.
(PDF)

S18 Table Statistical model results for Mean wing elevation (Y) across speeds. Model consists of intercept, U (X1) and U^2^ (X2). Individual (X4) is included as a random effect. Number of observations refers to the number of recorded flight sequences.
(PDF)

S19 Table AIC values for the models fitted to the kinematic data using a first (poly 1) and second (poly 2) order polynomial function with flight speed. The model with the lowest AIC was presented in this study.
(PDF)

S1 Data Data used in the figures found in the paper. Sheet “PIV-data” contains data obtained from flow field measurements and sheet Kin-data contains the data from kinematic measurements.
(XLSX)

S1 Code The Matlab code used to automatically identify the distalmost vortex and calculate the span and lift efficiency. The code also includes the subfunction AutoMask, which is used to automatically isolate the wake of the bird from the background flow, and used to reduce the noise in the measurements.
(DOCX)

S2 Code The Matlab code used to generate the PIV-related figures and statistical tests. (DOCX)

## Abbreviations

COT: cost of transport
MWE: mean wing elevation
PF: power factor
sPIV: stereo particle image velocimetry
