# Supplementary material for: Nightjars trade off cruising speed efficiency for hovering capabilities and link upstroke thrust production with restricted wing folding
Source: PLoS Biol. 2026 Sep 30;24(9):e3004023. doi: 10.1371/journal.pbio.3004023 (PMC13645225; doi:10.1371/journal.pbio.3004023)
Supplement: Supplementary file 1 [file pbio.3004023.s001.pdf]

Nightjars trade off cruising speed efficiency for hovering capabilities and link upstroke thrust production with restricted wing folding

L. Christoffer Johansson, Gabriel Norevik, Sonja Friman and Anders Hedenström

Supporting information

Material and methods

PIV settings

The PIV analysis was done in Davis 8 (LaVision, Göttingen, Germany). Before the analysis we preprocessed the images using subtract sliding background (size 8) to remove background image noise. For the velocity vector determination we used a decreasing box size, starting with 64x64, at 50% overlap and finishing with 3 times 32x32 boxes with 50% overlap. The box size is the size of the window used for cross-correlation to the other image of each image pair. We used the GPU option for the calculations, starting with a maximum shift of 5 pixels decreasing to 1 pixel in the final steps and used an initial shift of 3 pixels in the streamwise direction in the first round. These shift values determine where to look in the second image for the window sampled in the first image. Between each round we applied a two-times universal outlier detection, removing vectors deviating more than 2 standard deviations (SD) from their neighbors and reinserting the second-choice vector if deviating less than 3 SD from its neighbors. This is a statistical filter, removing vectors that differ significantly from its neighbors. After filling up empty spaces with interpolation we performed a smoothing using the "optimal" routine. After the analysis was done, the vector fields were post processed using a "Strongly remove and iteratively replace" filter with vectors removed if differing more than 2 SD from their neighbors and inserted if less than 3 SD. Empty spaces were filled by interpolation and a single "Optimal" smoothing was performed.

Results and discussion

We conducted a cross-correlation analysis (using xcorr in Matlab) between T and F_V over time and interpreted the lag between the curves, calculated as a percentage of the wingbeat period ($T_t = 1/f$), as the delay between the two forces. We found that the main thrust peak during the downstroke was time-shifted compared to the peak in vertical force, with the cross correlation showing an average delay of ~3.8% of the wingbeat period. We then tested if there was a correlation between T/F_V ($T = P/U_\infty$) and the delay, using a mixed

linear model with delay as dependent and T/F_V as independent with individual as a random factor and N_{wb} in the sequence as weight. We found a positive correlation between T/F_V and the delay between peak T and F_V (slope 16.0 CI 5.7-26.4, N=86, p =0.0028). The delay found between T and F_V may reflect a use of non-zero mean wing elevation relative to the horizontal plane, which has been proposed as a way to efficiently control the T to F_V ratio (51). This interpretation is further supported by a positive correlation between the T/F_V and the shift. A mean wing elevation below the horizontal plane will result in the peak F_V occurring before the wing reaches its peak angular velocity (due to the wing being closer to the horizontal plane earlier in the wingbeat) when T may be expected to reach a maximum. It will also facilitate wing-wing interaction at the transition between downstroke and upstroke, which may benefit T production (51). This is partly supported by the kinematics, where we see a decreasing trend in mean wing elevation with speed and at least for the highest speed a mean wing elevation substantially below the horizontal plane (S6g Fig).

In addition to the trends in mean span and vertical force efficiency (Fig 3d) we also found that span efficiency and vertical force efficiency behave very differently during the downstrokes (Fig 3e). While span efficiency is relatively constant throughout the downstroke, the vertical force efficiency peaks mid-downstroke (Fig 3e), which we consider to be quite illustrative. This reflects an often-ignored inherent cost of flapping flight. When flapping the wings to generate thrust, the wing also generates side forces that have no function and cancel between left and right wing, which results in accelerated air and adds to the cost of flight. However, the induced power is related to the induced velocity and the bird seems to maintain a constant, relatively high, span efficiency throughout the downstroke (Fig 3e), indicative of efficient aerodynamic lift generation rather than maintaining high vertical force efficiency. Particularly during the late phases of the downstroke can be important for thrust production (51) and then it makes sense to maintain a high span efficiency throughout the downstroke. We thus think that the tradeoff between span efficiency and vertical force efficiency needs further attention, especially in relation to thrust requirements.

The measured power is in relative agreement to the values predicted by the model for flapping flight (3)(Fig 3a). One difference relates to the minimum power speed (U_{mp}), which the model predicts to ~6 m/s while our data estimate it to ~9.3 m/s. The average flight speed during commuting flights have been estimated to 10 m/s or slightly above (61), which is higher than the maximum range speed indicated by the model (7.7 m/s). The discrepancy between model and measurements comes from the shape of the curves at high speeds, where the model predicts a faster increase in power with flight speed than the data (Fig 3a). This suggests further investigation of the body drag coefficient is needed, to improve model accuracy, since this is one of the main contributors to power at high flight

speed. At intermediate speeds we see double wing tip vortices while the induced power is a relatively large fraction of the total power and our measured power is indeed higher than the model predicts (Fig 3a). We also note that the measured power is lower than predicted at the lowest speed (Fig 3a), supporting our earlier suggestion that the birds have been selected for an efficient force generation at low speeds where the induced power dominates the costs, but could also be a result of wing-wake interaction (e.g. wake capture).

Regarding kinematics, many of our results are in line with previous studies. We find that stroke plane angle and body tilt angle mirror each other, where a pitch up of the body is accompanied by a more horizontally inclined stroke plane (Fig 4a). However, the change in stroke plane angle is twice the change in body tilt angle, indicating an ability to also control the flapping direction at the shoulder joint. We find a continuous decrease in flapping frequency with increasing flight speed (S6e Fig), but at the same time an increase in angular flapping amplitude (S6d Fig). The product of these parameters, the angular flapping velocity, is a measure of the flapping effort by the animal and is likely to correlate with the power required to fly (4,30,42). In this study the angular flapping velocity, however, has a minimum at a lower speed than indicated by our measured mechanical power (Figs 2b and 4b).

At the lowest flight speeds, we note a positive, rather high, tail tilt angle accompanied with a significant tail spread angle (S6 b, c Fig). Together, this would suggest a vertical force production by the tail, but we do not find distinct vortices suggestive of the expected force production (S2 Fig). However, in some of the wakes for the slowest speed we see a relatively week wake structure occurring below the upstroke wake that may indicate a pulsed vertical force production that may be attributed to the tail (S2 and S4 Figs). The lack of distinctive tail vortices at the lowest flight speed suggest that either the tail force generation is integrated with the flow over the wings and body in a way that cancels the vortices (i.e. interacts with wing root vortices) or that the wake is “deformed” from interactions in a way that prevents us from identifying the vortices. Alternatively, the tail may be inside the downwash generated by the wings and therefore have a lower angle of attack (and thereby a lower force generation) than suggested by the angle relative to the free stream flow. Further studies are required to determine which hypothesis is valid.

The species with the highest span ratio in Fig 5d (nightjars, swifts and hummingbirds) are relatively closely related, despite having very different flight styles and wing shapes. However, what they have in common is a relatively long hand wing and there is a possibility that their common ancestry has somehow prevented them from folding their hand wings during flight, rather than the hand wings not being folded for aerodynamic reasons. To

resolve this, a phylogenetically wider sample of species needs to be studied in future experiments (e.g. swallows and terns). An interesting option would be species, such as the doves and pigeons, which change span ratio substantially with flight speed (Fig 4d). This behavior suggests a change in the aerodynamic benefit of folding the wing during the upstroke e.g. when the wing tip velocity relative to the bird due to the sweeping motion becomes low compared to the forward flight speed.
