# Supplementary material for: Nightjars trade off cruising speed efficiency for hovering capabilities and link upstroke thrust production with restricted wing folding
Source: PLoS Biol. 2026 Sep 30;24(9):e3004023. doi: 10.1371/journal.pbio.3004023 (PMC13645225; doi:10.1371/journal.pbio.3004023)
Supplement: Supplementary file 2 [file pbio.3004023.s002.pdf]

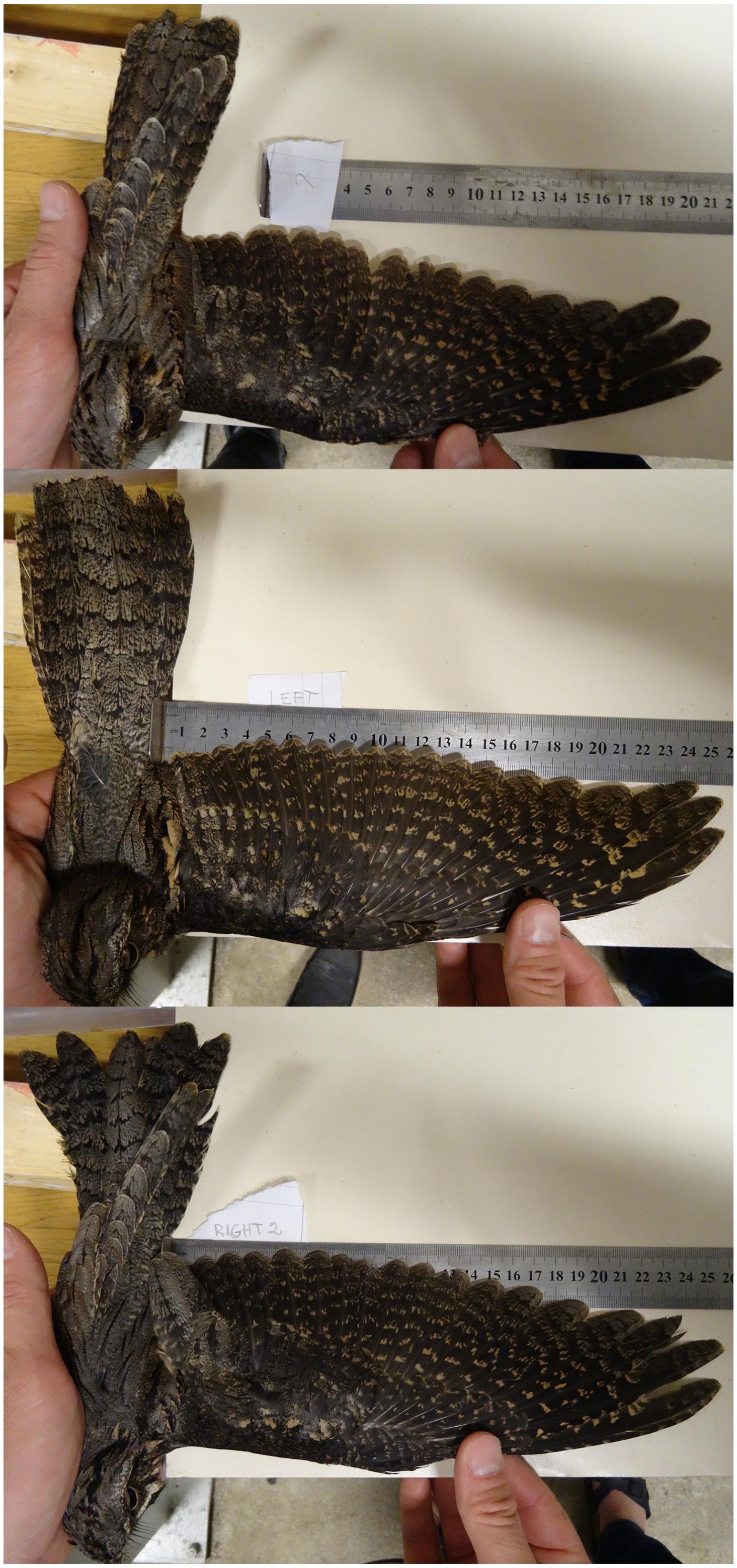


**S1 Figure.** Photos showing the wings of the nightjars used in this study, alfa (top), left (center) and Right2 (bottom).
