# Supplementary material for: Nightjars trade off cruising speed efficiency for hovering capabilities and link upstroke thrust production with restricted wing folding
Source: PLoS Biol. 2026 Sep 30;24(9):e3004023. doi: 10.1371/journal.pbio.3004023 (PMC13645225; doi:10.1371/journal.pbio.3004023)
Supplement: Supplementary file 3 [file pbio.3004023.s003.pdf]

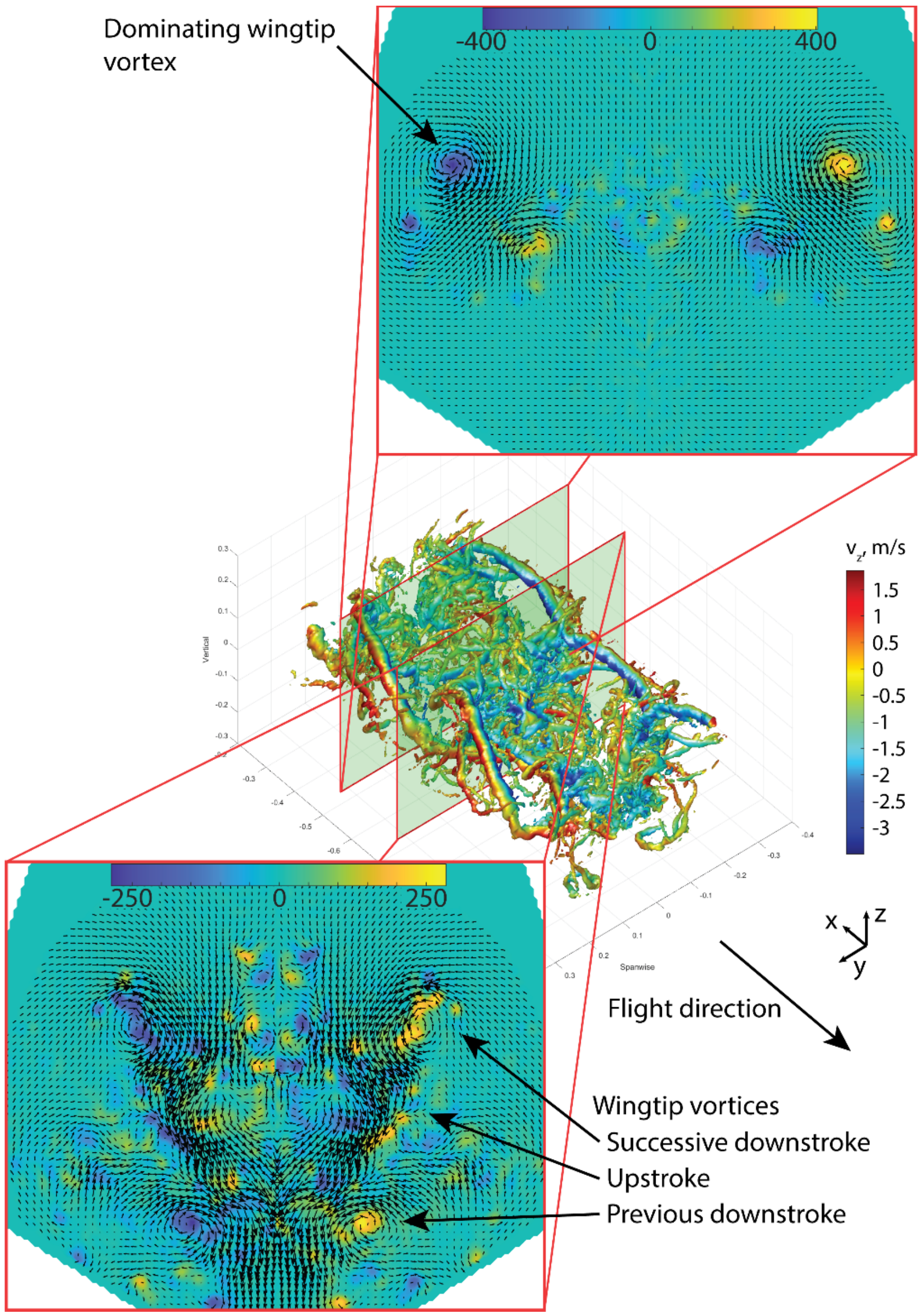

S2 Figure. Wake vortices shown as iso surfaces of total vorticity [s^{-1}] (iso value = 150), colored by downwash velocity of a sample sequence of a European nightjar flying at 2.6 m/s. Note the complex wake during the “upstroke” (lower inlet), cutting through two successive downstrokes and one upstroke and the weak flow in the body region during the downstroke (upper inlet). The inlets show in-plane velocity vectors on a background showing the streamwise vorticity with blue showing clockwise rotation and yellow/red counterclockwise rotation. Vectors are not scaled the same in inlets but are scaled to facilitate interpretation of the flow. Axes units are in m.
