# Supplementary material for: Nightjars trade off cruising speed efficiency for hovering capabilities and link upstroke thrust production with restricted wing folding
Source: PLoS Biol. 2026 Sep 30;24(9):e3004023. doi: 10.1371/journal.pbio.3004023 (PMC13645225; doi:10.1371/journal.pbio.3004023)
Supplement: Supplementary file 5 [file pbio.3004023.s005.pdf]

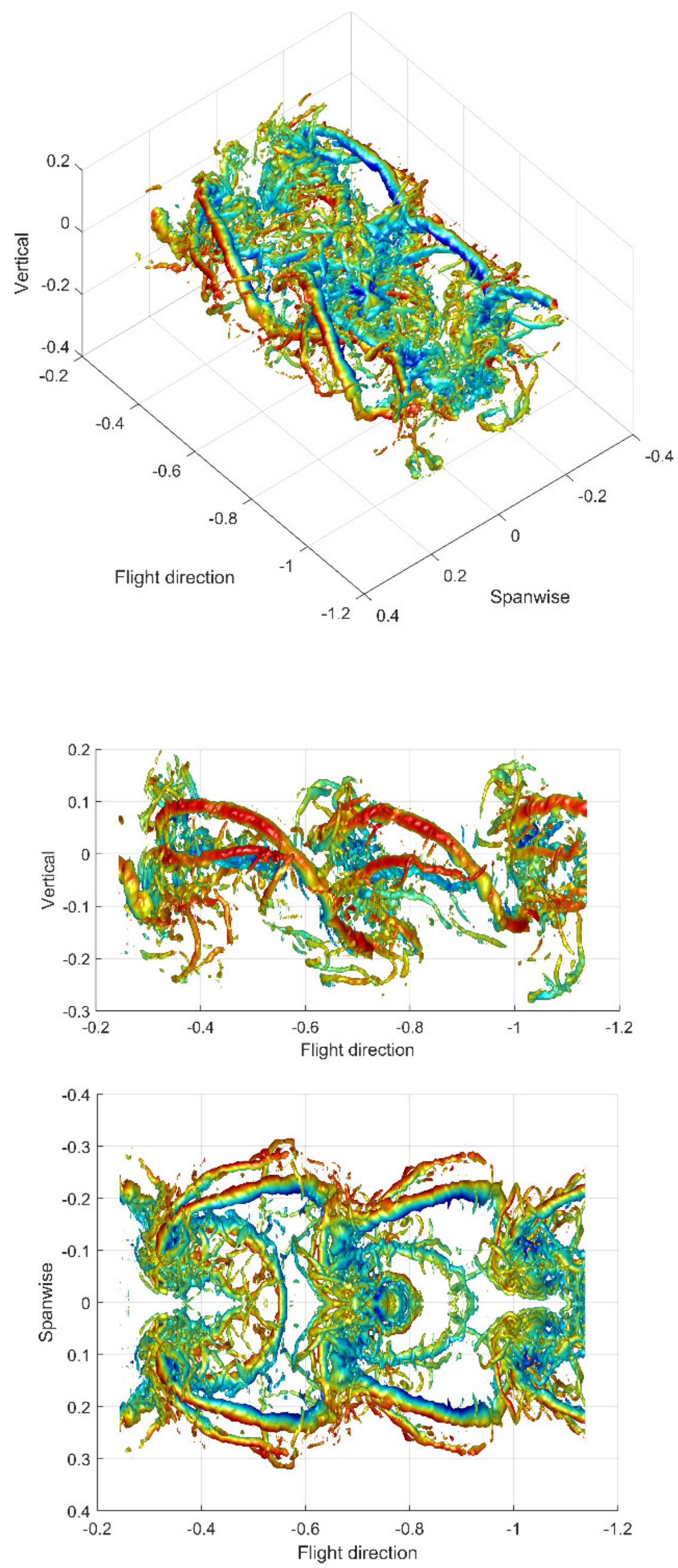

S4 Figure. Isosurfaces of the total vorticity [s^{-1}] (iso value 150) of the wake of a nightjar flying at 2.6 m/s. Oblique view (**a**), side view (**b**) and top view (**c**). The wake is mirrored in the center of the body and the bird is flying to the right. Axes units are in m.
