# Supplementary material for: Nightjars trade off cruising speed efficiency for hovering capabilities and link upstroke thrust production with restricted wing folding
Source: PLoS Biol. 2026 Sep 30;24(9):e3004023. doi: 10.1371/journal.pbio.3004023 (PMC13645225; doi:10.1371/journal.pbio.3004023)
Supplement: Supplementary file 6 [file pbio.3004023.s006.pdf]

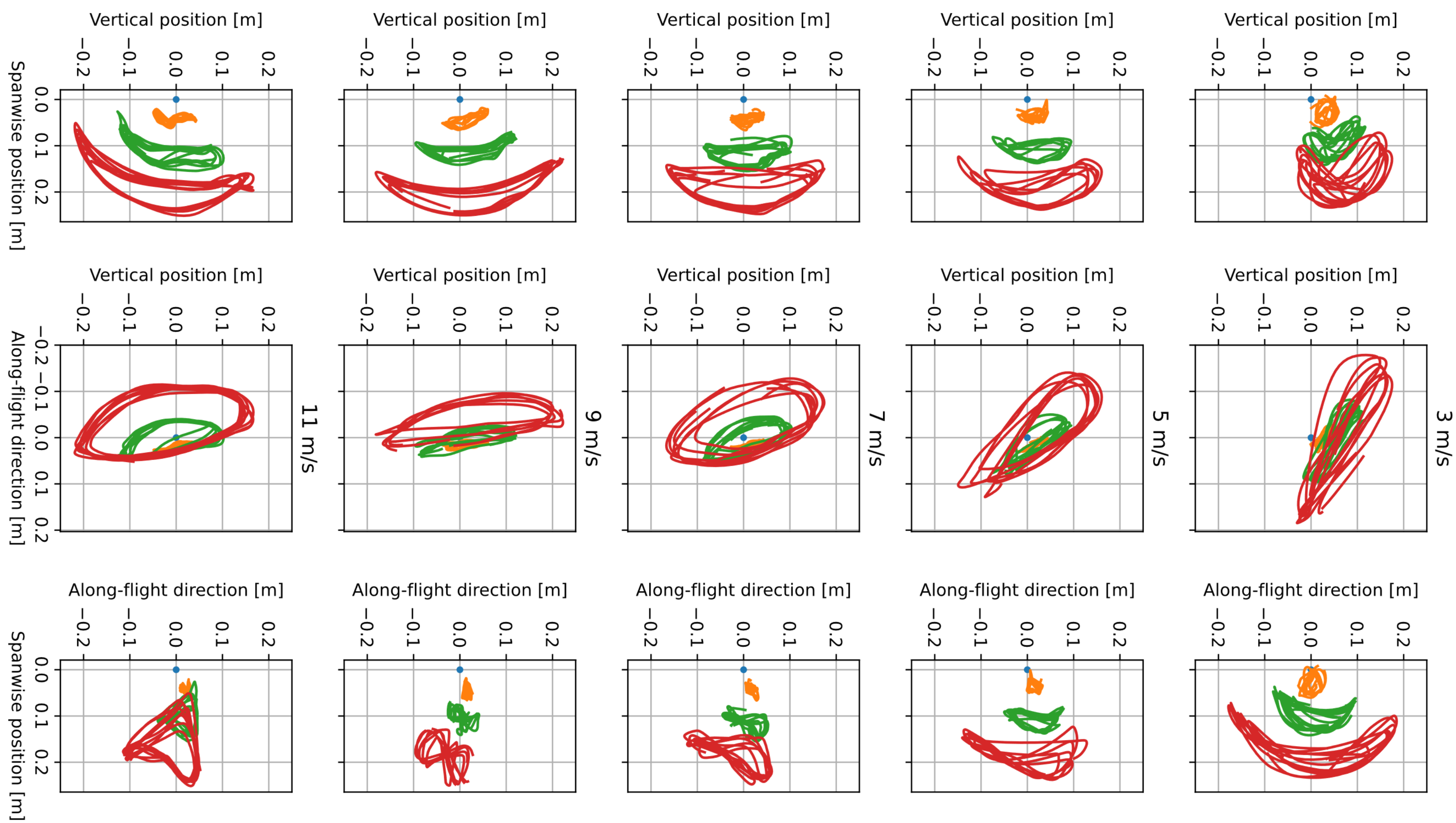

S5 Figure. Sample sequences of wingbeat motions across speeds in European nightjars. Tracks over frames for the wing tip (red), trailing edge (green), wrist (orange) relative to the shoulder (blue) as seen from the front (left column), the side (middle column) and below (right column), going from slow (top row) to fast flight (bottom row). Note the change in mean wing elevation across speed and the limited span ratio in the left column and the change in stroke plane in the middle column.
