# Supplementary material for: Nightjars trade off cruising speed efficiency for hovering capabilities and link upstroke thrust production with restricted wing folding
Source: PLoS Biol. 2026 Sep 30;24(9):e3004023. doi: 10.1371/journal.pbio.3004023 (PMC13645225; doi:10.1371/journal.pbio.3004023)
Supplement: Supplementary file 7 [file pbio.3004023.s007.pdf]

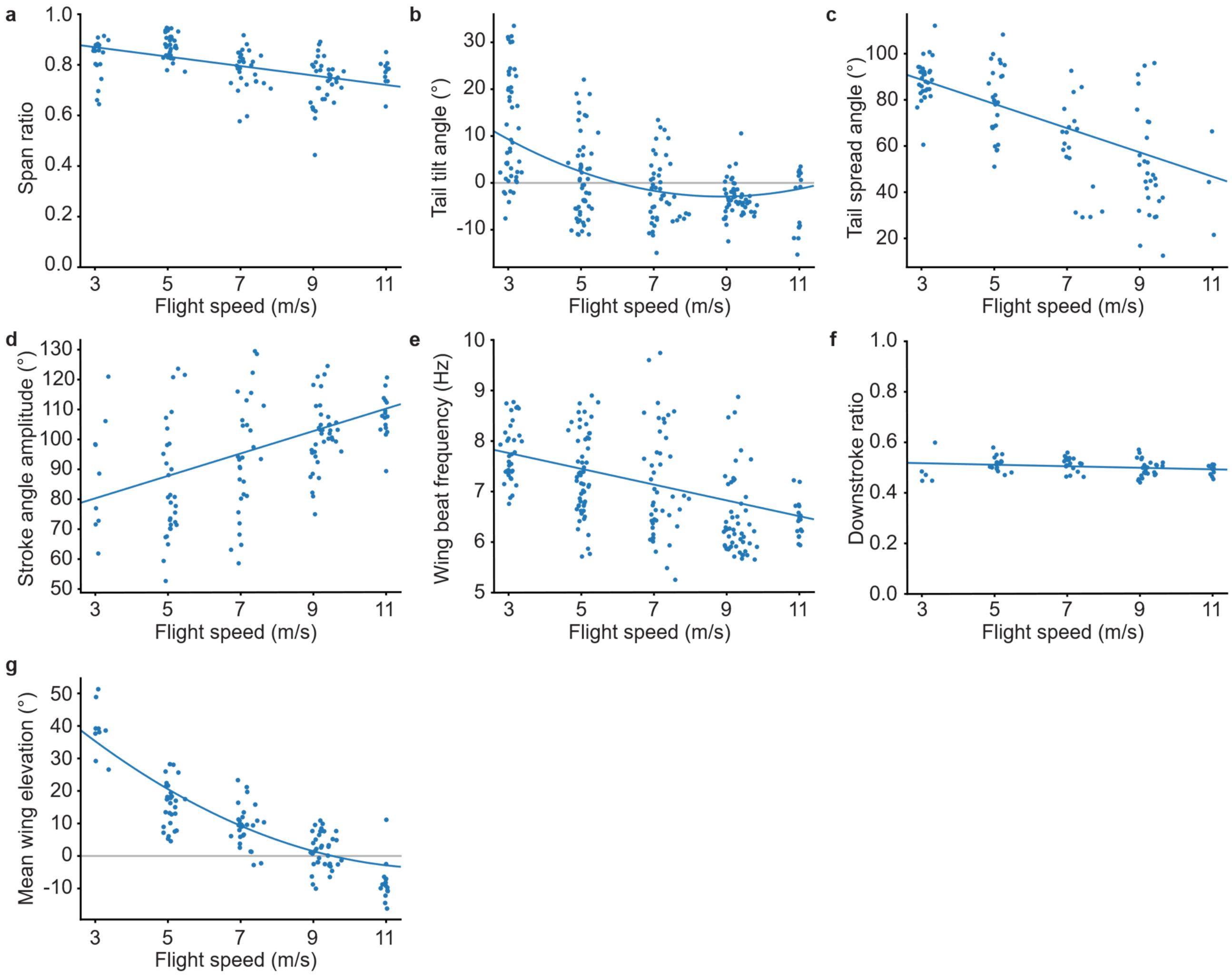

S6 Figure. Kinematics vary with flight speed. Nightjars show a high span ratio across flight speeds (**a**). The birds increased the tilt of the tail relative to the horizontal plane (tail tilt angle, [degrees]) (**b**) and tail area (tail spread angle, [degrees]) (**c**) at lower compared to higher speeds. Angular amplitude [degrees] increases with speed (**d**), while wingbeat frequency [Hz] decreases (**e**). Downstroke ratio is constant across flight speed (**f**). Mean wing elevation decreases [degrees] with increasing flight speed (**g**). For the statistics associated with the fitted lines we refer to S7 – S19 Tables. Data used to produce these figures can be found in S1 Data.
