# Supplementary material for: Nightjars trade off cruising speed efficiency for hovering capabilities and link upstroke thrust production with restricted wing folding
Source: PLoS Biol. 2026 Sep 30;24(9):e3004023. doi: 10.1371/journal.pbio.3004023 (PMC13645225; doi:10.1371/journal.pbio.3004023)
Supplement: Supplementary file 8 [file pbio.3004023.s008.pdf]

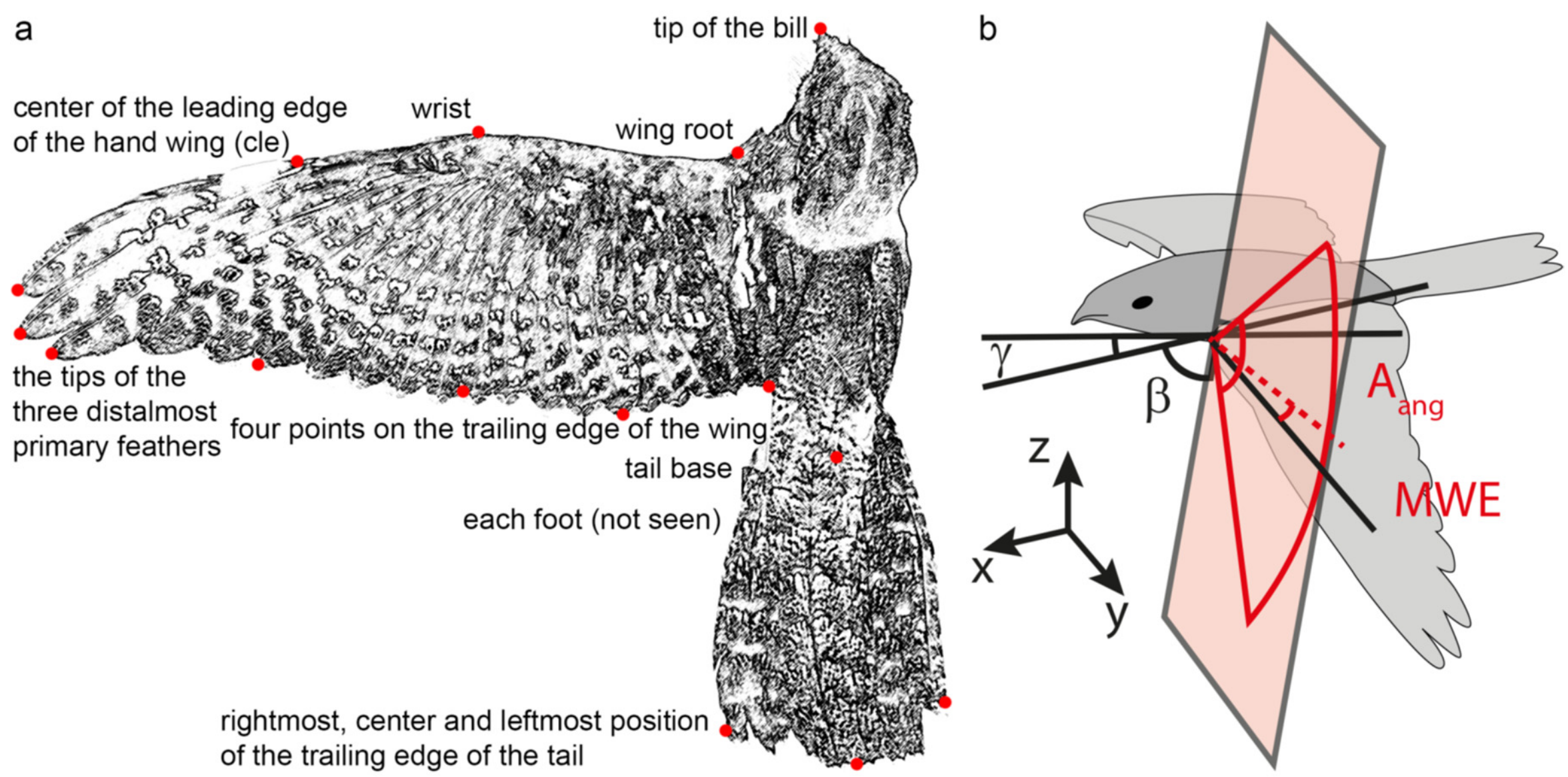

S7 Figure. Definition of the morphological points tracked for the kinematic analysis of nightjar flight (a). The two points on the feet are not seen in this view. (b) Definition of kinematic angles; stroke plane (β), body tilt angle (γ), angular amplitude (A_{ang}) and mean wing elevation (MWE).
