# Supplementary material for: Nightjars trade off cruising speed efficiency for hovering capabilities and link upstroke thrust production with restricted wing folding
Source: PLoS Biol. 2026 Sep 30;24(9):e3004023. doi: 10.1371/journal.pbio.3004023 (PMC13645225; doi:10.1371/journal.pbio.3004023)
Supplement: Supplementary file 9 [file pbio.3004023.s009.pdf]

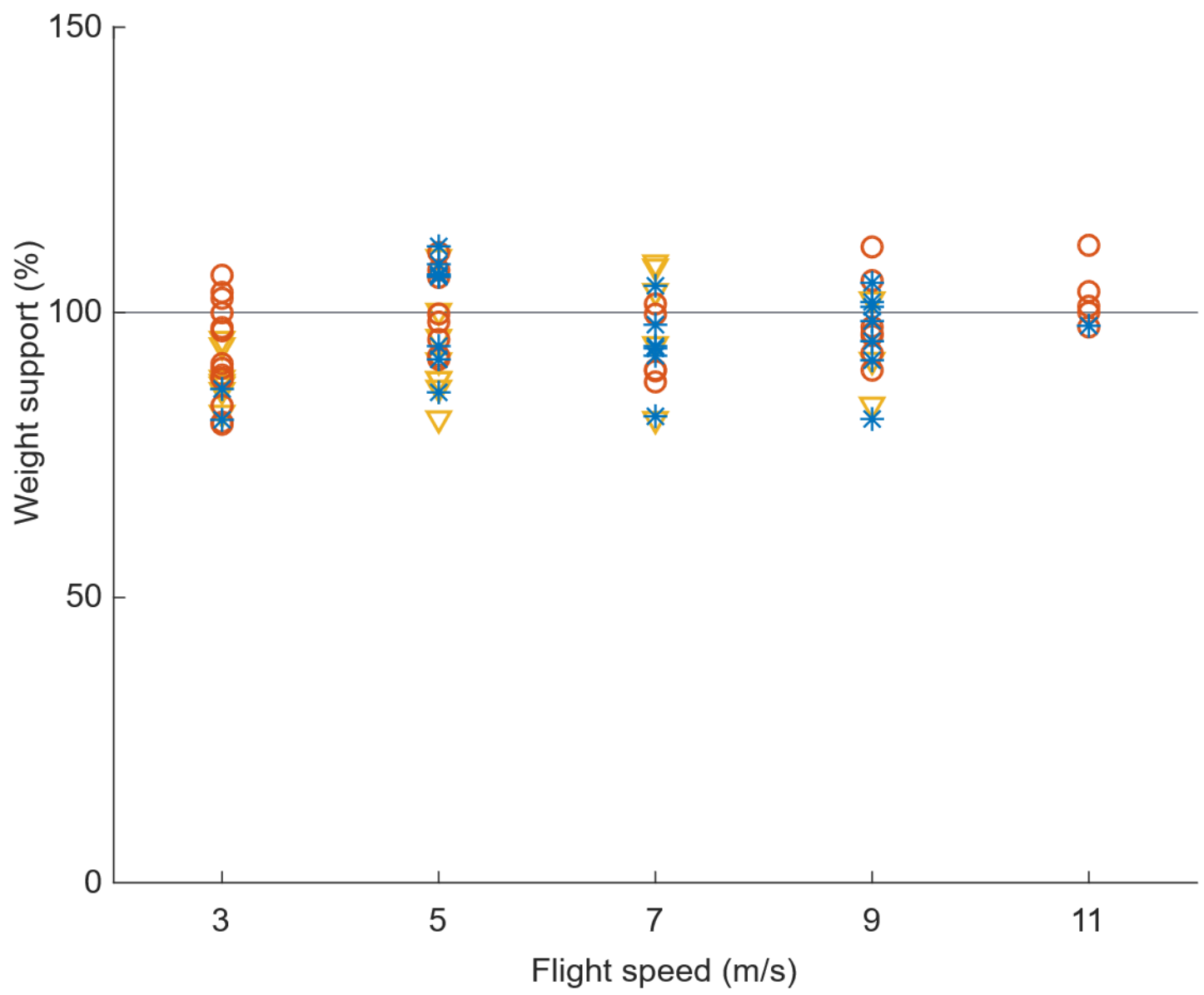

S8 Figure. Estimated weight support for the different individuals across flight speeds. Asterix, individual “Left”; circle, “Right2”; triangle, “Alfa”. Each point represents the mean value for one sequence (1-7 wingbeats). Data used to produce this figure can be found in S1 Data.
