# Supplementary material for: Nightjars trade off cruising speed efficiency for hovering capabilities and link upstroke thrust production with restricted wing folding
Source: PLoS Biol. 2026 Sep 30;24(9):e3004023. doi: 10.1371/journal.pbio.3004023 (PMC13645225; doi:10.1371/journal.pbio.3004023)
Supplement: Supplementary file 10 [file pbio.3004023.s010.pdf]

S1 Table. Morphometric information for the three European nightjars included in the analysis.

Individual	Mass (kg)	Wing span (m)	Wing area (m^2)	Wing loading (N/m^2)	Aspect ratio
Left	0.0579	0.57	0.0422	13.4	7.6
Right2	0.0789	0.56	0.0442	17.5	7.1
Alfa	0.0775	0.56	0.0422	18.9	7.7
