# Supplementary material for: Nightjars trade off cruising speed efficiency for hovering capabilities and link upstroke thrust production with restricted wing folding
Source: PLoS Biol. 2026 Sep 30;24(9):e3004023. doi: 10.1371/journal.pbio.3004023 (PMC13645225; doi:10.1371/journal.pbio.3004023)
Supplement: Supplementary file 11 [file pbio.3004023.s011.pdf]

S2 Table. Statistical model results for P/F_v (Y) across speeds. Model assumes constant profile power, induced power varying with 1/U (X1) and parasitic power varying with U^3 (X2). Individual (X4) is included as a random effect and data are weighed by number of wingbeats in the sequence. Number of observations refers to the number of recorded flight sequences.

Linear mixed-effects model fit by REML

Model information:

Number of observations	86
Fixed effects coefficients	3
Random effects coefficients	9
Covariance parameters	4

Formula:

Y ~ 1 + X1 + X2 + (1 | X4) + (X1 | X4) + (X2 | X4)

Model fit statistics:

AIC	BIC	LogLikelihood	Deviance
-116.16	-99.224	65.078	-130.16

Fixed effects coefficients (95% CIs):

Name	Estimate	SE	tStat	DF	pValue	Lower	Upper
Intercept	0.524	0.0571	9.19	83	2.78e-14	0.411	0.638
X1	0.897	0.147	6.10	83	3.17e-08	0.604	1.19
X2	3.92e-05	4.49e-05	0.87	83	0.385	-5.01e-05	0.000129

Random effects covariance parameters (95% CIs):

Name1	Name2	Type	Estimate	Lower	Upper
Group: X4 (3 Levels)					
Intercept	Intercept	std	0.0626	0.0213	0.184
Group: X4 (3 Levels)					
X1	X1	std	4.84e-06	NaN	NaN
Group: X4 (3 Levels)					
X2	X2	std	1.24e-05	1.06e-05	1.45e-05
Group: Error					
Res Std			0.157	0.135	0.183
