# Supplementary material for: Nightjars trade off cruising speed efficiency for hovering capabilities and link upstroke thrust production with restricted wing folding
Source: PLoS Biol. 2026 Sep 30;24(9):e3004023. doi: 10.1371/journal.pbio.3004023 (PMC13645225; doi:10.1371/journal.pbio.3004023)
Supplement: Supplementary file 12 [file pbio.3004023.s012.pdf]

S3 Table. Statistical model results for COT (Y) across speeds. Model assumes constant profile drag, induced drag varying with $1/U^2$ (X1) and parasitic drag varying with U^2 (X2). Individual (X4) is included as a random effect and data are weighed by number of wingbeats in the sequence. Number of observations refers to the number of recorded flight sequences.

Linear mixed-effects model fit by REML

Model information:

Number of observations	86
Fixed effects coefficients	3
Random effects coefficients	9
Covariance parameters	4

Formula:

Y ~ 1 + X1 + X2 + (1 | X4) + (X1 | X4) + (X2 | X4)

Model fit statistics

AIC	BIC	LogLikelihood	Deviance
-369.02	-352.09	191.51	-383.02

Fixed effects coefficients (95% CIs):

Name	Estimate	SE	tStat	DF	pValue	Lower	Upper
Intercept	0.0750	0.0104	7.19	83	2.65e-10	0.0542	0.0957
X1	1.78	0.0676	26.3	83	4.44e-42	1.65	1.92
X2	-0.000276	0.000108	-2.56	83	0.0123	-0.000490	-6.14e-05

Random effects covariance parameters (95% CIs):

Name1	Name2	Type	Estimate	Lower	Upper
Group: X4 (3 Levels)					
Intercept	Intercept	std	0.0114	0.00371	0.0353
Group: X4 (3 Levels)					
X1	X1	std	5.44e-10	NaN	NaN
Group: X4 (3 Levels)					
X2	X2	std	3.20e-14	NaN	NaN
Group: Error					
Res Std			0.0357	0.0306	0.0417
