# Supplementary material for: Nightjars trade off cruising speed efficiency for hovering capabilities and link upstroke thrust production with restricted wing folding
Source: PLoS Biol. 2026 Sep 30;24(9):e3004023. doi: 10.1371/journal.pbio.3004023 (PMC13645225; doi:10.1371/journal.pbio.3004023)
Supplement: Supplementary file 13 [file pbio.3004023.s013.pdf]

S4 Table. Statistical model results for PF (Y) across speeds. Model consists of intercept, U (X1) and U^2 (X2). Individual (X4) is included as a random effect and data are weighed by number of wingbeats in the sequence. Number of observations refers to the number of recorded flight sequences.

Linear mixed-effects model fit by REML

Model information:

Number of observations	86
Fixed effects coefficients	3
Random effects coefficients	9
Covariance parameters	4

Formula:

Y ~ 1 + X1 + X2 + (1 | X4) + (X1 | X4) + (X2 | X4)

Model fit statistics:

AIC	BIC	LogLikelihood	Deviance
280.71	297.64	-133.36	266.71

Fixed effects coefficients (95% CIs):

Name	Estimate	SE	tStat	DF	pValue	Lower	Upper
Intercept	4.24	0.648	6.54	83	4.80e-09	2.95	5.53
X1	0.969	0.239	4.05	83	0.000117	0.492	1.45
X2	-0.0606	0.0185	-3.27	83	0.00155	-0.0974	-0.0238

Random effects covariance parameters (95% CIs):

Name1	Name2	Type	Estimate	Lower	Upper
Group: X4 (3 Levels)					
Intercept	Intercept	std	0.230	0.00247	21.5
Group: X4 (3 Levels)					
X1	X1	std	0.114	0.0294	0.442
Group: X4 (3 Levels)					
X2	X2	std	3.23e-12	NaN	NaN
Group: Error					
Res Std			1.72	1.47	2.01
