# Supplementary material for: Nightjars trade off cruising speed efficiency for hovering capabilities and link upstroke thrust production with restricted wing folding
Source: PLoS Biol. 2026 Sep 30;24(9):e3004023. doi: 10.1371/journal.pbio.3004023 (PMC13645225; doi:10.1371/journal.pbio.3004023)
Supplement: Supplementary file 14 [file pbio.3004023.s014.pdf]

S5 Table. Statistical model results for e_i (Y) across speeds. Model consists of intercept and exp(U) (X1). Individual (X4) is included as a random effect and data are weighed by number of wingbeats in the sequence. Number of observations refers to the number of recorded flight sequences.

Linear mixed-effects model fit by REML

Model information:

Number of observations	78
Fixed effects coefficients	2
Random effects coefficients	3
Covariance parameters	2

Formula:

Y1 ~ 1 + X1 + (1 | X4)

Model fit statistics:

AIC	BIC	LogLikelihood	Deviance
-201.12	-191.8	104.56	-209.12

Fixed effects coefficients (95% CIs):

Name	Estimate	SE	tStat	DF	pValue	Lower	Upper
Intercept	0.743	0.00590	126	76	5.50e-90	0.731	0.755
X1	-2.72e-06	4.10e-07	-6.63	76	4.27e-09	-3.53e-06	-1.90e-06

Random effects covariance parameters (95% CIs):

Name1	Name2	Type	Estimate	Lower	Upper
Group: X4 (3 Levels)					
Intercept	Intercept	std	9.47e-18	NaN	NaN
Group: Error					
Res Std			0.0853	0.0728	0.100
