# Supplementary material for: Nightjars trade off cruising speed efficiency for hovering capabilities and link upstroke thrust production with restricted wing folding
Source: PLoS Biol. 2026 Sep 30;24(9):e3004023. doi: 10.1371/journal.pbio.3004023 (PMC13645225; doi:10.1371/journal.pbio.3004023)
Supplement: Supplementary file 15 [file pbio.3004023.s015.pdf]

S6 Table. Statistical model results for e_{Fv} (Y) across speeds. Model consists of intercept and exp(U) (X1). Individual (X4) is included as a random effect and data are weighed by number of wingbeats in the sequence. Number of observations refers to the number of recorded flight sequences.

Linear mixed-effects model fit by REML

Model in�ormation:

Number of observations	78
Fixed effects coefficients	2
Random effects coefficients	3
Covariance parameters	2

Formula:

Y2 ~ 1 + X1 + (1 | X4)

Model fit statistics:

AIC	BIC	LogLikelihood	Deviance
-107.72	-98.398	57.861	-115.72

Fixed effects coefficients (95% CIs):

Name	Estimate	SE	tStat	DF	pValue	Lower	Upper
Intercept	0.495	0.0109	45.4	76	8.10e-57	0.474	0.517
X1	-2.79e-06	7.57e-07	-3.67	76	0.000424	-4.30e-06	-1.28e-06

Random effects covariance parameters (95% CIs):

Name1	Name2	Type	Estimate	Lower	Upper
Group: X4 (3 Levels)					
Intercept	Intercept	std	0	NaN	NaN
Group: Error					
Res Std			0.158	0.134	0.185
