# Supplementary material for: Nightjars trade off cruising speed efficiency for hovering capabilities and link upstroke thrust production with restricted wing folding
Source: PLoS Biol. 2026 Sep 30;24(9):e3004023. doi: 10.1371/journal.pbio.3004023 (PMC13645225; doi:10.1371/journal.pbio.3004023)
Supplement: Supplementary file 16 [file pbio.3004023.s016.pdf]

S7 Table. Statistical model results for Body tilt angle (Y) across speeds. Model consists of intercept, U (X1) and U^2 (X2). Individual (X4) is included as a random effect. Number of observations refers to the number of recorded flight sequences.

Linear mixed-effects model fit by REML

Model information

Number of observations	212
Fixed effects coefficients	3
Random effects coefficients	3
Covariance parameters	2

Formula:

Y ~ 1 + X1 + X2 + (1 | X4)

Model fit statistics:

AIC	BIC	LogLikelihood	Deviance
1361.5	1378.2	-675.73	1351.5

Fixed effects coefficients (95% CIs):

Name	Estimate	SE	tStat	DF	pValue	Lower	Upper
Intercept	54.5	3.06	17.8	209	8.34e-44	48.5	60.5
X1	-7.87	0.947	-8.32	209	1.16e-14	-9.74	-6.01
X2	0.378	0.0699	5.41	209	1.75e-07	0.240	0.516

Random effects covariance parameters (95% CIs):

Name1	Name2	Type	Estimate	Lower	Upper
Group: X4 (3 Levels)					
Intercept	Intercept	std	1.76	0.566	5.47
Group: Error					
Res Std			5.77	5.24	6.36
