# Supplementary material for: Nightjars trade off cruising speed efficiency for hovering capabilities and link upstroke thrust production with restricted wing folding
Source: PLoS Biol. 2026 Sep 30;24(9):e3004023. doi: 10.1371/journal.pbio.3004023 (PMC13645225; doi:10.1371/journal.pbio.3004023)
Supplement: Supplementary file 17 [file pbio.3004023.s017.pdf]

S8 Table. Statistical model results for Stroke plane angle (Y) across speeds. Model consists of intercept, U (X1) and U^2 (X2). Individual (X4) is included as a random effect. Number of observations refers to the number of recorded flight sequences.

Linear mixed-effects model fit by REML

Model in�ormation:

Number of observations	122
Fixed effects coefficients	3
Random effects coefficients	3
Covariance parameters	2

Formula:

Y ~ 1 + X1 + X2 + (1 | X4)

Model fit statistics:

AIC	BIC	LogLikelihood	Deviance
747.16	761.06	-368.58	737.16

Fixed effects coefficients (95% CIs):

Name	Estimate	SE	tStat	DF	pValue	Lower	Upper
Intercept	11.3	4.54	2.49	119	0.0141	2.32	20.3
X1	12.5	1.25	9.97	119	2.20e-17	9.98	14.9
X2	-0.562	0.0850	-6.61	119	1.13e-09	-0.731	-0.394

Random effects covariance parameters (95% CIs):

Name1	Name2	Type	Estimate	Lower	Upper
Group: X4 (3 Levels)					
Intercept	Intercept	std	2.55	0.877	7.42
Group: Error					
Res Std			4.84	4.26	5.50
