# Supplementary material for: Nightjars trade off cruising speed efficiency for hovering capabilities and link upstroke thrust production with restricted wing folding
Source: PLoS Biol. 2026 Sep 30;24(9):e3004023. doi: 10.1371/journal.pbio.3004023 (PMC13645225; doi:10.1371/journal.pbio.3004023)
Supplement: Supplementary file 18 [file pbio.3004023.s018.pdf]

S9 Table. Statistical model results for Tail tilt angle (Y) across speeds. Model consists of intercept, U (X1) and U^2 (X2). Individual (X4) is included as a random effect. Number of observations refers to the number of recorded flight sequences.

Linear mixed-effects model fit by REML

Model information:

Number of observations	210
Fixed effects coefficients	3
Random effects coefficients	3
Covariance parameters	2

Formula:

Y ~ 1 + X1 + X2 + (1 | X4)

Model fit statistics:

AIC	BIC	LogLikelihood	Deviance
1444.6	1461.3	-717.3	1434.6

Fixed effects coefficients (95% CIs):

Name	Estimate	SE	tStat	DF	pValue	Lower	Upper
Intercept	27.3	4.49	6.09	207	5.36e-09	18.5	36.2
X1	-6.76	1.21	-5.61	207	6.55e-08	-9.13	-4.38
X2	0.374	0.0895	4.17	207	4.43e-05	0.197	0.550

Random effects covariance parameters (95% CIs):

Name1	Name2	Type	Estimate	Lower	Upper
Group: X4 (3 Levels)					
Intercept	Intercept	std	4.5367	1.6476	12.492
Group: Error					
Res Std			7.2334	6.5661	7.9686
