# Supplementary material for: Nightjars trade off cruising speed efficiency for hovering capabilities and link upstroke thrust production with restricted wing folding
Source: PLoS Biol. 2026 Sep 30;24(9):e3004023. doi: 10.1371/journal.pbio.3004023 (PMC13645225; doi:10.1371/journal.pbio.3004023)
Supplement: Supplementary file 19 [file pbio.3004023.s019.pdf]

S10 Table. Statistical model results for Tail spread angle (Y) across speeds. Model consists of intercept and U (X1). Individual (X4) is included as a random effect. Number of observations refers to the number of recorded flight sequences.

Linear mixed-effects model fit by REML

Model information:

Number of observations	108
Fixed effects coefficients	2
Random effects coefficients	3
Covariance parameters	2

Formula:

Y ~ 1 + X1 + (1 | X4)

Model fit statistics:

AIC	BIC	LogLikelihood	Deviance
875.68	886.34	-433.84	867.68

Fixed effects coefficients (95% CIs):

Name	Estimate	SE	tStat	DF	pValue	Lower	Upper
Intercept	107	8.14	13.1	106	6.35e-24	90.6	123
X1	-5.73	0.518	-11.1	106	2.30e-19	-6.76	-4.70

Random effects covariance parameters (95% CIs):

Name1	Name2	Type	Estimate	Lower	Upper
Group: X4 (3 Levels)					
Intercept	Intercept	std	12.813	4.6518	35.291
Group: Error					
Res Std			13.303	11.612	15.239
