# Supplementary material for: Nightjars trade off cruising speed efficiency for hovering capabilities and link upstroke thrust production with restricted wing folding
Source: PLoS Biol. 2026 Sep 30;24(9):e3004023. doi: 10.1371/journal.pbio.3004023 (PMC13645225; doi:10.1371/journal.pbio.3004023)
Supplement: Supplementary file 20 [file pbio.3004023.s020.pdf]

S11 Table. Statistical model results for Stroke angle amplitude (Y) across speeds. Model consists of intercept and U (X1). Individual (X4) is included as a random effect. Number of observations refers to the number of recorded flight sequences.

Linear mixed-effects model fit by REML

Model information:

Number of observations	122
Fixed effects coefficients	2
Random effects coefficients	3
Covariance parameters	2

Formula:

Y ~ 1 + X1 + (1 | X4)

Model fit statistics:

AIC	BIC	LogLikelihood	Deviance
1006.5	1017.7	-499.27	998.53

Fixed effects coefficients (95% CIs):

Name	Estimate	SE	tStat	DF	pValue	Lower	Upper
Intercept	69.4	6.38	10.9	120	1.41e-19	56.7	82
X1	3.70	0.564	6.55	120	1.48e-09	2.58	4.82

Random effects covariance parameters (95% CIs):

Name1	Name2	Type	Estimate	Lower	Upper
Group: X4 (3 Levels)					
Intercept	Intercept	std	8.00	2.68	23.9
Group: Error					
Res Std			14.5	12.7	16.5
