# Supplementary material for: Nightjars trade off cruising speed efficiency for hovering capabilities and link upstroke thrust production with restricted wing folding
Source: PLoS Biol. 2026 Sep 30;24(9):e3004023. doi: 10.1371/journal.pbio.3004023 (PMC13645225; doi:10.1371/journal.pbio.3004023)
Supplement: Supplementary file 21 [file pbio.3004023.s021.pdf]

S12 Table. Statistical model results for Wingbeat frequency (Y) across speeds. Model consists of intercept and U (X1). Individual (X4) is included as a random effect. Number of observations refers to the number of recorded flight sequences.

Linear mixed-effects model fit by REML

Model information:

Number of observations	212
Fixed effects coefficients	2
Random effects coefficients	3
Covariance parameters	2

Formula:

Y ~ 1 + X1 + (1 | X4)

Model fit statistics:

AIC	BIC	LogLikelihood	Deviance
435.42	448.81	-213.71	427.42

Fixed effects coefficients (95% CIs):

Name	Estimate	SE	tStat	DF	pValue	Lower	Upper
Intercept	8.24	0.408	20.2	210	3.92e-51	7.43	9.04
X1	-0.159	0.0177	-8.97	210	1.71e-16	-0.193	-0.124

Random effects covariance parameters (95% CIs):

Name1	Name2	Type	Estimate	Lower	Upper
Group: X4 (3 Levels)					
Intercept	Intercept	std	0.672	0.248	1.82
Group: Error					
Res Std			0.637	0.578	0.701
