# Supplementary material for: Nightjars trade off cruising speed efficiency for hovering capabilities and link upstroke thrust production with restricted wing folding
Source: PLoS Biol. 2026 Sep 30;24(9):e3004023. doi: 10.1371/journal.pbio.3004023 (PMC13645225; doi:10.1371/journal.pbio.3004023)
Supplement: Supplementary file 22 [file pbio.3004023.s022.pdf]

S13 Table. Statistical model results for Downstroke ratio (Y) across speeds. Model consists of intercept and U (X1). Individual (X4) is included as a random effect. Number of observations refers to the number of recorded flight sequences.

Linear mixed-effects model fit by REML

Model information:

Number of observations	79
Fixed effects coefficients	2
Random effects coefficients	3
Covariance parameters	2

Formula:

Y ~ 1 + X1 + (1 | X4)

Model fit statistics:

AIC	BIC	LogLikelihood	Deviance
-291.71	-282.33	149.85	-299.71

Fixed effects coefficients (95% CIs):

Name	Estimate	SE	tStat	DF	pValue	Lower	Upper
Intercept	0.527	0.0128	41.2	77	3.18e-54	0.501	0.552
X1	-0.00308	0.00162	-1.90	77	0.0608	-0.00630	0.000143

Random effects covariance parameters (95% CIs):

Name1	Name2	Type	Estimate	Lower	Upper
Group: X4 (3 Levels)					
Intercept	Intercept	std	3.59e-18	NaN	NaN
Group: Error					
Res Std			0.0323	0.0276	0.0378
