# Supplementary material for: Nightjars trade off cruising speed efficiency for hovering capabilities and link upstroke thrust production with restricted wing folding
Source: PLoS Biol. 2026 Sep 30;24(9):e3004023. doi: 10.1371/journal.pbio.3004023 (PMC13645225; doi:10.1371/journal.pbio.3004023)
Supplement: Supplementary file 24 [file pbio.3004023.s024.pdf]

S15 Table. Statistical model results for Span ratio (Y) across speeds. Model consists of intercept and U (X1). Individual (X4) is included as a random effect. Number of observations refers to the number of recorded flight sequences.

Linear mixed-effects model fit by REML

Model information:

Number of observations	129
Fixed effects coefficients	2
Random effects coefficients	3
Covariance parameters	2

Formula:

Y ~ 1 + X1 + (1 | X4)

Model fit statistics:

AIC	BIC	LogLikelihood	Deviance
-262.57	-251.19	135.28	-270.57

Fixed effects coefficients (95% CIs):

Name	Estimate	SE	tStat	DF	pValue	Lower	Upper
Intercept	0.925	0.0245	37.8	127	6.28e-71	0.877	0.974
X1	-0.0184	0.00291	-6.32	127	4.04e-09	-0.0242	-0.0127

Random effects covariance parameters (95% CIs):

Name1	Name2	Type	Estimate	Lower	Upper
Group: X4 (3 Levels)					
Intercept	Intercept	std	0.0204	0.00563	0.0741
Group: Error					
Res Std			0.0789	0.0697	0.0893
