# Supplementary material for: Nightjars trade off cruising speed efficiency for hovering capabilities and link upstroke thrust production with restricted wing folding
Source: PLoS Biol. 2026 Sep 30;24(9):e3004023. doi: 10.1371/journal.pbio.3004023 (PMC13645225; doi:10.1371/journal.pbio.3004023)
Supplement: Supplementary file 25 [file pbio.3004023.s025.pdf]

S16 Table. Statistical model results for Sweep angle DS (Y) across speeds. Model consists of intercept, U (X1) and U^2 (X2). Individual (X4) is included as a random effect. Number of observations refers to the number of recorded flight sequences.

Linear mixed-effects model fit by REML

Model information:

Number of observations	100
Fixed effects coefficients	3
Random effects coefficients	3
Covariance parameters	2

Formula:

Y ~ 1 + X1 + X2 + (1 | X4)

Model fit statistics:

AIC	BIC	LogLikelihood	Deviance
704.44	717.32	-347.22	694.44

Fixed effects coefficients (95% CIs):

Name	Estimate	SE	tStat	DF	pValue	Lower	Upper
Intercept	54.1	7.46	7.26	97	9.78e-11	39.3	68.9
X1	6.63	2.19	3.03	97	0.00313	2.29	11.0
X2	-0.306	0.150	-2.04	97	0.0445	-0.605	-0.00774

Random effects covariance parameters (95% CIs):

Name1	Name2	Type	Estimate	Lower	Upper
Group: X4 (3 Levels)					
Intercept	Intercept	std	0	NaN	NaN
Group: Error					
Res Std			7.88	6.84	9.07
