# Supplementary material for: Nightjars trade off cruising speed efficiency for hovering capabilities and link upstroke thrust production with restricted wing folding
Source: PLoS Biol. 2026 Sep 30;24(9):e3004023. doi: 10.1371/journal.pbio.3004023 (PMC13645225; doi:10.1371/journal.pbio.3004023)
Supplement: Supplementary file 26 [file pbio.3004023.s026.pdf]

S17 Table. Statistical model results for Sweep angle US (Y) across speeds. Model consists of intercept, U (X1) and U^2 (X2). Individual (X4) is included as a random effect. Number of observations refers to the number of recorded flight sequences.

Linear mixed-effects model fit by REML

Model in?ormation:

Number of observations	91
Fixed effects coefficients	3
Random effects coefficients	3
Covariance parameters	2

Formula:

Y ~ 1 + X1 + X2 + (1 | X4)

Model fit statistics:

AIC	BIC	LogLikelihood	Deviance
741.67	754.06	-365.84	731.67

Fixed effects coefficients (95% CIs):

Name	Estimate	SE	tStat	DF	pValue	Lower	Upper
Intercept	24.8	14.5	1.71	88	0.0914	-4.07	53.6
X1	15.3	3.98	3.85	88	0.000220	7.43	23.3
X2	-1.04	0.271	-3.86	88	0.000215	-1.58	-0.507

Random effects covariance parameters (95% CIs):

Name1	Name2	Type	Estimate	Lower	Upper
Group: X4 (3 Levels)					
Intercept	Intercept	std	7.42	2.51	21.9
Group: Error					
Res Std			13.6	11.7	15.8
