# Supplementary material for: Nightjars trade off cruising speed efficiency for hovering capabilities and link upstroke thrust production with restricted wing folding
Source: PLoS Biol. 2026 Sep 30;24(9):e3004023. doi: 10.1371/journal.pbio.3004023 (PMC13645225; doi:10.1371/journal.pbio.3004023)
Supplement: Supplementary file 27 [file pbio.3004023.s027.pdf]

S18 Table. Statistical model results for Mean wing elevation (Y) across speeds. Model consists of intercept, U (X1) and U^2 (X2). Individual (X4) is included as a random effect. Number of observations refers to the number of recorded flight sequences.

Linear mixed-effects model fit by REML

Model information:

Number of observations	122
Fixed effects coefficients	3
Random effects coefficients	3
Covariance parameters	2

Formula:

Y ~ 1 + X1 + X2 + (1 | X4)

Model fit statistics:

AIC	BIC	LogLikelihood	Deviance
803.75	817.65	-396.88	793.75

Fixed effects coefficients (95% CIs):

Name	Estimate	SE	tStat	DF	pValue	Lower	Upper
Intercept	63.9	5.99	10.7	119	4.64e-19	52.1	75.8
X1	-10.8	1.58	-6.87	119	3.20e-10	-14.0	-7.71
X2	0.434	0.107	4.03	119	9.69e-05	0.221	0.646

Random effects covariance parameters (95% CIs):

Name1	Name2	Type	Estimate	Lower	Upper
Group: X4 (3 Levels)					
Intercept	Intercept	std	4.37	1.53	12.5
Group: Error					
Res Std			6.11	5.37	6.94
