# Supplementary material for: Nightjars trade off cruising speed efficiency for hovering capabilities and link upstroke thrust production with restricted wing folding
Source: PLoS Biol. 2026 Sep 30;24(9):e3004023. doi: 10.1371/journal.pbio.3004023 (PMC13645225; doi:10.1371/journal.pbio.3004023)
Supplement: Supplementary file 28 [file pbio.3004023.s028.pdf]

S19 Table. AIC values for the models fitted to the kinematic data using a first (poly 1) and second (poly 2) order polynomial function with flight speed. The model with the lowest AIC was presented in this study.

AIC	Linear	Quadratic
Body tilt angle	1383.4	**1361.5**
Down stroke ratio	**-291.71**	-281.23
Folding angle DS	704.59	**704.44**
Folding angle US	752.78	**741.67**
Mean DS angular velocity	1094.8	**1074.7**
Mean wing elevation	814.4	**803.75**
Span ratio	**-262.57**	-249.11
Stroke angle amplitude	**1006.5**	1006.7
Stroke plane angle	779.58	**747.16**
Tail spread angle	**875.68**	878.13
Tail tilt angle	1456.4	**1444.6**
Wingbeat frequency	**435.42**	442.99
