# Supplementary material for: Nightjars trade off cruising speed efficiency for hovering capabilities and link upstroke thrust production with restricted wing folding
Source: PLoS Biol. 2026 Sep 30;24(9):e3004023. doi: 10.1371/journal.pbio.3004023 (PMC13645225; doi:10.1371/journal.pbio.3004023)
Supplement: Supplementary file 29 [file pbio.3004023.s030.docx]

function spaneff

% This code is used to estimate the span efficiency, based on the flow behind a single wing during
% forward flapping flight in a wind tunnel, using flow fields obtained through Particle image
% velocimetry. It does that by automatically identifying the distalmost, strong, tip vortex and using the
% induced velocities along a line connecting the center of that vortex to the center of the body wake.
% It starts by loading a file containing a struct variable with fields containing the x, y, z coordinates
% of velocity vectors in the fields vx, vy, vz as well as vorticity wx, wy, wz. The coordinate system has % its origin at the center of the body wake in the xy-plane. In addition, the struct contains a mask
% (AmaskW), previously generated to isolate the vortices associated with the wake from the
% background flow.

% Corrdinate definisions: x is the spanwise direction in the Treffts plane of the wind tunnel and
% increases along the second dimension of the 3D data matrix. y is the vertical direction in the Treffts
% plane of the wind tunnel and decreases along the first dimension of the 3D data matrix. z is in the
% free stream direction of the wind tunnel and decreases along the third dimension of the 3D data
% matrix.

cd('N:\Files done')

seqs = dir;

for k=3:size(seqs,1)

if seqs(k).isdir

cd('N:\Files done')

cd(['N:\Files done\',seqs(k).name])

files = dir('*_DO.mat');

load(files(1).name)

X = DataOut.x(:,:,1);

Y = DataOut.y(:,:,1);

MASK = X<=0;

for m = 1:size(DataOut.x,3)

th = 0.8;

wztemp = DataOut.wz(:,:,m).*DataOut.AmaskW(:,:,m); %Use in plane vorticity

wztemps = smoothdata2(wztemp,"gaussian",21);

wztempz(isnan(wztemps)) = 0;

TF = islocalmax2(abs(wztemps.*MASK),MinProminence=max(abs(wztemps.*MASK),[],'all','omitnan')*th);

j=0;

while (sum(TF,'all')<3 || sum(TF,'all')>5) && j<100

if sum(TF,'all')>5

th = th*1.031;

elseif sum(TF,'all')<3

th = th*0.971;

end

TF = islocalmax2(abs(wztemps.*MASK),MinProminence=max(abs(wztemps.*MASK),[],'all','omitnan')*th);

j=j+1;

end

b = find(TF);

Dist = (X(TF).^2+Y(TF).^2).^0.5;

[B,I] = sort(Dist.^2.*abs(-wztemps(TF)));

B2 = b(I);

Tips(m) = B2(end);

end

ft = fittype( 'sin3' );

opts = fitoptions( 'Method', 'NonlinearLeastSquares' );

opts.Display = 'Off';

opts.Lower = [-Inf 0 -Inf -Inf 0 -Inf -Inf 0 -Inf];

opts.Robust = 'Bisquare';

Dist = (X(Tips).^2+Y(Tips).^2).^0.5;

sedata.Dist = Dist;

tempt = 1:length(Tips);

tempX = Dist-mean(Dist,'all');

[fitresult, gof] = fit(tempt', tempX', ft, opts );

XX = feval(fitresult,tempt');

stderr = std(Dist./(XX+mean(Dist,'all'))');

a = Dist./(XX+mean(Dist,'all'))'-1>2*stderr;

b = Dist./(XX+mean(Dist,'all'))'-1<-2*stderr;

c = a+b;

tempt(c==1) = [];

tempX(c==1) = [];

[fitresult, gof] = fit(tempt', tempX', ft, opts);

XX2 = feval(fitresult,tempt');

stderr = std(tempX-XX2');

a = (tempX-XX2')>2*stderr;

b = (tempX-XX2')<-2*stderr;

c = a+b;

tempt(c==1) = [];

tempX(c==1) = [];

Ytips = Y(Tips);

Xtips = X(Tips);

Ytipssm = csaps(tempt,Ytips(tempt),[],1:length(Ytips));

Xtipssm = csaps(tempt,Xtips(tempt),[],1:length(Xtips));

theta = atan(Ytipssm./Xtipssm);

sm = csaps(tempt,Ytips(tempt),1e-3);

sedata.Y = fnval(sm,1:length(Ytips));

ds = fnval(fnder(sm),1:length(Ytips));

sedata.dsY = ds;

[fitresult, gof] = fit(tempt', Ytipssm(tempt)'-mean(Ytipssm(tempt)','all'), ft, opts);

Ysm = feval(fitresult,1:size(Tips,2)');

ds2 = fnval(fnder(spline(1:size(Tips,2),Ysm)),1:length(Ytips));

sedata.dsSinY = ds2;

Ds = csaps(1:size(Tips,2),Dist,1e-3);

Dds = fnval(1:size(Tips,2),fnder(Ds));

sedata.dsDist = Dds;

dx = DataOut.x(1,2,1)-DataOut.x(1,1,1);

for m = 1:size(DataOut.x,3)

R = [cos(theta(m)) -sin(theta(m)); sin(theta(m)) cos(theta(m))];

tiprot = [Xtipssm(m),Ytipssm(m)]*R;

vx = DataOut.vx(:,:,m);

vy = DataOut.vy(:,:,m);

xyrot = [X(:),Y(:)]*R;

vrot = [vx(:),vy(:)]*R;

xi = sign(tiprot(1))*(0: dx:abs(tiprot(1))+ dx*0.9);

F = scatteredInterpolant(xyrot(:,1), xyrot(:,2), vrot(:,2));

vi(m,1:size(xi,2)) = F(xi, zeros(1,size(xi,2)));

Pi(m) = sum(abs(xi(2)-xi(1))*abs(vi(m,1:size(xi,2))).^3);

L = -sum(sign(vi(m,1:size(xi,2))).*vi(m,1:size(xi,2)).^2)/length(xi);

vm = sign(L)*(abs(L))^0.5;

Pii(m) = (abs(xi(2)-xi(1))*abs(vm).^3)*size(xi,2);

ei(m) = Pii(m)/Pi(m);

vmL = vm*cos(theta(m));

PiiL(m) = (abs(xi(2)-xi(1))*vmL.^3)*size(xi,2);

eiL(m) = PiiL(m)/Pi(m);

end

sedata.eimed = median(ei(ds2<0|ds<0));

sedata.eiLmed = median(eiL(ds2<0|ds<0));

% Save data

FileName = [seqs(k).name(6:20),'_DO_se.mat'];

save(FileName,'Pii','Pi','PiiL','ei','eiL','sedata')

end

clearvars DataOut f

end

function Amask = AutoMask(wtot,Th,MOS)

% This code isolates the vortices in the wake of a flying animal from background noise. It applies a
% threshold (Th) and a minimum objects size (MOS) to total vorticity (wtot) in a 3D matrix. Size of
% strel objects may need to be adjusted, depending on the resolution and complexity of the wake.

m1 = imbinarize(wtot,Th);

m2 = bwareaopen(m1,MOS,26);

SEC = strel('sphere',5);

m3 = imclose(m2,SEC);

SE1 = strel('cuboid',[2,2,2]);

m4 = imerode(m3,SE1);

SE2 = strel('cuboid',[4,4,4]);

Amask = imdilate(m4,SE2);

Amask = imclose(Amask,SEC);

SE3 = strel('cuboid',[3,3,3]);

Amask = imerode(Amask,SE3);

function [wbf, Ldff, Ldfp, Tdff, Tdfp, timeShift] = LTtiming(Lt,Tt)

% This code uses the timeseries of lift and thrust to determine the dominant frequences in the two
% datasets and the two additional dominant frequencies of the thrust. In addition, it estimates the time % shift between the peaks of lift and thrust.

% Lift analysis

D = Lt-mean(Lt,'omitnan');

D(isnan(D)) = [];

D = smooth(D,7);

Y = fft(D,2048);

Pyy = Y.* conj(Y) / 2048;

f = 640*(0:126)/2048;

[m, pos] = max(Pyy);

wbf = f(pos);

[p loc] = findpeaks(Pyy(1:127),f,'SortStr','descend','NPeaks',3);

Ldff(1,1:length(loc)) = loc;

Ldfp(1,1:length(p)) = p/p(1);

clear D Y Pyy m pos p loc

% Thrust analysis

D = Tt-mean(Tt,'omitnan');

D(isnan(D)) = [];

D = smooth(D,7);

Y = fft(D,2048);

Pyy = Y.* conj(Y) / 2048;

[m, pos] = max(Pyy);

[p loc] = findpeaks(Pyy(1:127),f,'SortStr','descend','NPeaks',3);

Tdff(1,1:length(loc)) = loc;

Tdfp(1,1:length(p)) = p/p(1);

Tdfp(1,1) = p(1);

% Compute cross-correlation and find the lag with maximum correlation

timeSeries1 = Lt;

timeSeries2 = Tt

timeSeries1(isnan(timeSeries1)) = [];

timeSeries2(isnan(timeSeries2)) = [];

[correlation, lags] = xcorr(timeSeries1, timeSeries2, round(length(timeSeries1)/Nwb(i)/2));

[~, maxIndex] = max(correlation);

timeShift(i,1) = lags(maxIndex);
