# Supplementary material for: Nightjars trade off cruising speed efficiency for hovering capabilities and link upstroke thrust production with restricted wing folding
Source: PLoS Biol. 2026 Sep 30;24(9):e3004023. doi: 10.1371/journal.pbio.3004023 (PMC13645225; doi:10.1371/journal.pbio.3004023)
Supplement: Supplementary file 30 [file pbio.3004023.s031.docx]

% function NJDataAnalysisRed

clear all

cd('C:\Data')

T = readtable('PIVData.xlsx');

Ind = zeros(size(T,1),1);

I = table2array(T(:,1));

for i = 1:size(I,1)

if I{i,1}(1,2) == 'L'

Ind(i)=1;

elseif I{i,1}(1,2) == 'A'

Ind(i)=3;

elseif I{i,1}(1,2) == 'R'

Ind(i)=2;

end

end

M = table2array(T(:,2)); %Mass

U = table2array(T(:,3)); %Speed

Nwb = table2array(T(:,4)); %Number of wingbeats

L = table2array(T(:,5)); % Lift

P = table2array(T(:,6)); %Power

COT = table2array(T(:,7)); % Cost of transport

PF = table2array(T(:,8)); % Power factor

ei = table2array(T(:,9)); % Span efficiency

eiL = table2array(T(:,10)); % Lift efficiency

T1L1 = table2array(T(:,11)); % Main frequency of thrust/Main frequency of lift

T2T1 = table2array(T(:,12)); % Second frequency of thrust/Main frequency of thrust

figure(3)

clf

plot(U(Ind==1), L(Ind==1,:)./(M(Ind==1)*9.81)*100,'*')

hold on

plot(U(Ind==2), L(Ind==2,:)./(M(Ind==2)*9.81)*100,'o')

plot(U(Ind==3), L(Ind==3,:)./(M(Ind==3)*9.81)*100,'v')

set(gca,'ylim',[0, 150])

set(gca,'xlim',[2, 12])

xlabel('Speed, m/s')

ylabel('Lift % of weight')

% Sample data

X1 = 1./U; % Continuous variable 1

X2 = U.^3; % Continuous variable 2

X3 = U.^2;

X4 = categorical(Ind); % Nominal variable with three states

X5 = Nwb;

Y = P./L; % Continuous response variable

% Create a table for the data

Sdata = table(X1, X2, X3, X4, X5, Y);

% Fit the GLM

glmModel = fitlme(Sdata, 'Y ~ X1 + X2 + (1|X4)+ (X1-1|X4)+ (X2-1|X4)','FitMethod','REML','Weights',X5);

% Display the results

disp(glmModel);

% Extract fixed-effect results

txt = evalc('disp(glmModel)');

% Optional: replace repeated spaces with tabs

txt_tab = regexprep(txt, ' {2,}', '\t');

% Write to text file

% cd('C:\Statistics')

% fid = fopen('Power_LME_results.txt','w');

% fprintf(fid, '%s', txt_tab);

% fclose(fid);

figure(7)

clf

hold on

U0 = 2.5:0.1:11.5;

Est = glmModel.Coefficients{1,2}+glmModel.Coefficients{2,2}./U0+glmModel.Coefficients{3,2}*U0.^3;

plot(U0,Est,'-b')

Sdata2 = table(1./U0', U0.^3', categorical(zeros(length(U0),1)),...

'VariableNames', glmModel.PredictorNames);

ypred = predict(glmModel, Sdata2);

plot(U0,ypred,'-b')

plot(U,P./L,'ob')

set(gca,'ylim',[0 1.2])

xlabel('Speed, m/s')

ylabel('Weight specific power, W/N')

figure(166)

clf

hold on

Ubox = [categorical(round(U));categorical(round(U))];

fbox = [T1L1;T2T1];

C = [categorical(ones(size(U)));categorical(ones(size(U))*2)];

boxchart(Ubox, fbox, 'GroupByColor', C);

xlabel('Speed, m/s')

ylabel('Multiple of wingbeat frequency')

figure(22)

clf

hold on

plot(U,P./(U.*L),'ok')

xlabel('Speed, m/s')

ylabel('Efficiency. COT')

X1 = U.^-2; % Continuous variable 1

X2 = U.^2; % Continuous variable 2

X4 = categorical(Ind); % Nominal variable with three states

X5 = Nwb;

Y = P./(U.*L); % Continuous response variable

% Create a table for the data

Sdata = table(X1, X2, X4, Y);

% Fit the GLM

glmModel = fitlme(Sdata, 'Y ~ X1 + X2 + (1|X4)+ (X1-1|X4)+ (X2-1|X4)','FitMethod','REML','Weights',X5);

disp(glmModel);

U0 = 2.5:0.1:11.5;

Est = glmModel.Coefficients{1,2}+glmModel.Coefficients{2,2}.*U0.^-2+glmModel.Coefficients{3,2}*U0.^2;

plot(U0,Est,'-k')

txt = evalc('disp(glmModel)');

% Optional: replace repeated spaces with tabs

txt_tab = regexprep(txt, ' {2,}', '\t');

% Write to text file

% cd('C:\Statistics')

% fid = fopen('COT_LME_results.txt','w');

% fprintf(fid, '%s', txt_tab);

% fclose(fid);

% Results from afpt, in R Klein Heerenbrink M, Johansson LC, Hedenström A. Power of the wingbeat: modelling the...

% effects of flapping wings in vertebrate flight. Proc R Soc Math Phys Eng Sci. 2015 May;471(2177):20140952. ...

% doi:10.1098/rspa.2014.0952

Umodel = 1:0.1:16;

Pmodel = [1.7971854 1.6401352 1.5081897 1.3956291 1.2983831 1.2134770 1.1386856 1.0723097 1.0130279 0.9597952 0.9117731...

0.8682794 0.8287522 0.7927239 0.7598014 0.7296513 0.7019886 0.6765681 0.6531774 0.6316314 0.6117682 0.5934456...

0.5765379 0.5609339 0.5465345 0.5332518 0.5209825 0.5095082 0.4987814 0.4887858 0.4795026 0.4709108 0.4629885...

0.4557130 0.4490615 0.4430114 0.4375405 0.4326274 0.4282513 0.4243924 0.4210320 0.4181522 0.4157361 0.4137678...

0.4122326 0.4111164 0.4104061 0.4100896 0.4101554 0.4105931 0.4113926 0.4125448 0.4140412 0.4158737 0.4180351...

0.4205184 0.4233172 0.4264258 0.4298385 0.4335504 0.4375566 0.4418529 0.4464352 0.4512997 0.4564429 0.4618617...

0.4675531 0.4735143 0.4797427 0.4862361 0.4929923 0.5000092 0.5072849 0.5148179 0.5226065 0.5306492 0.5389447...

0.5474917 0.5562892 0.5653360 0.5746311 0.5841736 0.5939627 0.6039975 0.6142772 0.6248012 0.6355687 0.6465808...

0.6578399 0.6693457 0.6810977 0.6930959 0.7053412 0.7178319 0.7305682 0.7435503 0.7567781 0.7702516 0.7839709...

0.7979359 0.8121469 0.8266039 0.8413070 0.8562565 0.8714525 0.8868951 0.9025847 0.9185215 0.9347056 0.9511375...

0.9678172 0.9847453 1.0019219 1.0193474 1.0370221 1.0549464 1.0731205 1.0915450 1.1102201 1.1291462 1.1483238...

1.1677532 1.1874348 1.2073691 1.2275564 1.2479973 1.2634592 1.2828312 1.3024013 1.3221690 1.3421340 1.3622960...

1.3826548 1.4032100 1.4239615 1.4449090 1.4660524 1.4873914 1.5089259 1.5306558 1.5525811 1.5747016 1.5970174...

1.6195284 1.6422347 1.6651364 1.6882335 1.7115262 1.7350147 1.7586992 1.7825800];

figure(7)

plot(Umodel(16:106),Pmodel(16:106)/(0.072*9.81),'-k')

figure(31)

clf

plot(U,ei,'ok')

hold on

plot(U,eiL,'ob')

set(gca,'ylim',[0 1])

xlabel('Speed, m/s')

ylabel('Span efficiency and lift efficiency')

X1 = exp(U); % Continuous variable 1

X2 = U.^2; % Continuous variable 2

X3 = U.^3; % Continuous variable 2

X4 = U.^4; % Continuous variable 2

X5 = categorical(Ind); % Nominal variable with three states

X6 = Nwb;

Y1 = ei; % Continuous response variable

Y2 = (eiL); % Continuous response variable

% Create a table for the data

Sdata = table(X1, X2, X3, X4, X5, Y1, Y2);

% Fit the GLM

glmModel1 = fitlme(Sdata, 'Y1 ~ X1 + (1|X5)','FitMethod','REML','Weights',X6);

glmModel2 = fitlme(Sdata, 'Y2 ~ X1 + (1|X5)','FitMethod','REML','Weights',X6);

% Display the results

disp(glmModel1);

disp(glmModel2);

Est1 = (glmModel1.Coefficients{1,2}+glmModel1.Coefficients{2,2}*exp(U0));%+glmModel1.Coefficients{3,2}*U0.^2);

Est2 = (glmModel2.Coefficients{1,2}+glmModel2.Coefficients{2,2}*exp(U0));%+glmModel2.Coefficients{3,2}*U0.^2);

plot(U0,Est1,'-k')

plot(U0,Est2,'-b')

txt = evalc('disp(glmModel1)');

% Optional: replace repeated spaces with tabs

txt_tab = regexprep(txt, ' {2,}', '\t');

% Write to text file

% cd('C:\Statistics')

% fid = fopen('ei_LME_results.txt','w');

% fprintf(fid, '%s', txt_tab);

% fclose(fid);

txt = evalc('disp(glmModel2)');

% Optional: replace repeated spaces with tabs

txt_tab = regexprep(txt, ' {2,}', '\t');

% Write to text file

% cd('C:\Statistics')

% fid = fopen('eFv_LME_results.txt','w');

% fprintf(fid, '%s', txt_tab);

% fclose(fid);

WA = zeros(size(Ind));

WA(Ind==1) = 0.04225; % Left

WA(Ind==2) = 0.04028; % alfa

WA(Ind==3) = 0.0442; % Right2

F = ((P./U).^2 + L.^2).^0.5;

CF = F./(0.5*1.2*U.^2.*WA);

CP = P./(0.5*1.2*U.^3.*WA);

figure(33)

clf

plot(U,CF.^1.5./CP,'ok')

hold on

xlabel('Speed, m/s')

ylabel('Power factor')

set(gca,'ylim',[0 15])

X1 = U; % Continuous variable 1

X2 = U.^2; % Continuous variable 2

X4 = categorical(Ind); % Nominal variable with three states

X5 = Nwb;

Y = (CF.^1.5)./CP; % Continuous response variable

% Create a table for the data

Sdata = table(X1, X2, X4, Y);

% Fit the GLM

glmModel = fitlme(Sdata, 'Y ~ X1 + X2 + (1|X4)+ (X1-1|X4)+ (X2-1|X4)','FitMethod','REML','Weights',X5);

disp(glmModel);

U0 = 2.5:0.1:11.5;

Est = glmModel.Coefficients{1,2}+glmModel.Coefficients{2,2}.*U0+glmModel.Coefficients{3,2}*U0.^2;

plot(U0,Est,'-k')

txt = evalc('disp(glmModel)');

% Optional: replace repeated spaces with tabs

txt_tab = regexprep(txt, ' {2,}', '\t');

% Write to text file

% cd('C:\Statistics')

% fid = fopen('PF_LME_results.txt','w');

% fprintf(fid, '%s', txt_tab);

% fclose(fid);
